# Three AP2/ERF family members modulate flavonoid synthesis by regulating type IV chalcone isomerase in citrus

**DOI:** 10.1111/pbi.13494

**Published:** 2020-11-27

**Authors:** Chenning Zhao, Xiaojuan Liu, Qin Gong, Jinping Cao, Wanxia Shen, Xueren Yin, Donald Grierson, Bo Zhang, Changjie Xu, Xian Li, Kunsong Chen, Chongde Sun

**Affiliations:** ^1^ Laboratory of Fruit Quality Biology/Zhejiang Provincial Key Laboratory of Horticultural Plant Integrative Biology/The State Agriculture Ministry Laboratory of Horticultural Plant Growth Development and Quality Improvement Zhejiang University Hangzhou China; ^2^ Citrus Research Institute Southwest University/Chinese Academy of Agricultural Sciences Chongqing China; ^3^ Division of Plant and Crop Sciences School of Biosciences University of Nottingham Loughborough UK

**Keywords:** citrus, flavanone, flavone, type IV chalcone isomerase, DREB, RAV

## Abstract

Flavanones and flavones are excellent source of bioactive compounds but the molecular basis of their highly efficient production remains elusive. Chalcone isomerase (CHI) family proteins play essential roles in flavonoid biosynthesis but little are known about the transcription factors controlling their gene expression. Here, we identified a type IV CHI (designated as CitCHIL1) from citrus which enhances the accumulation of citrus flavanones and flavones (CFLs). CitCHIL1 participates in a CFL biosynthetic metabolon and assists the cyclization of naringenin chalcone to (*2S*)‐naringenin, which leads to the efficient influx of substrates to chalcone synthase (CHS) and improves the catalytic efficiency of CHS. Overexpressing *CitCHIL1* in *Citrus* and *Arabidopsis* significantly increased flavonoid content and RNA interference‐induced silencing of CitCHIL1 in citrus led to a 43% reduction in CFL content. Three AP2/ERF transcription factors were identified as positive regulators of the *CitCHIL1* expression. Of these, two dehydration‐responsive element binding (DREB) proteins, CitERF32 and CitERF33, activated the transcription by directly binding to the CGCCGC motif in the promoter, while CitRAV1 (RAV: related to ABI3/VP1) formed a transcription complex with CitERF33 that strongly enhanced the activation efficiency and flavonoid accumulation. These results not only illustrate the specific function that CitCHIL1 executes in CFL biosynthesis but also reveal a new DREB‐RAV transcriptional complex regulating flavonoid production.

## Introduction

Flavonoids represent a range of plant metabolites which exhibit diverse structures, ubiquitous distribution and play critical roles in plant growth and adaption such as coloration, signalling and response to biotic/abiotic stresses (Brunetti *et al*., 2018; Moore *et al*., 2014). According to their chemical structures, they can be divided into diverse subclasses including flavanones, flavones, flavonols, isoflavonoids, anthocyanidins and proanthocyanidins (PAs) (Winkel‐Shirley, 2001). Although each class of flavonoids is synthesized from a distinct branch of the biosynthetic pathway and is under separate regulation, flavanones produced by stepwise catalysis of chalcone synthase (CHS) and chalcone isomerase (CHI) are the essential precursors of other flavonoids. In the flavonoid pathway, CHS catalyses the rate‐limiting step and CHI guarantees the rapid formation of biologically active (*S*)‐isomer. The regulatory mechanisms governing the genes for these early crucial steps are intriguing, complex and diverse, depending on species and flavonoids of interest.

The CHI superfamily is an ancient family and comprises four types of proteins (type I‐IV). Type I and type II CHI are *bona fide* CHIs with enzymatic cyclization activity (Ralston *et al*., 2005). Type III CHI is fatty‐acid‐binding protein (FAP) devoid of catalytic activities, which is the prototype of other CHI‐fold proteins (Ngaki *et al*., 2012). Type IV CHI is an evolutionary intermediate between FAP and *bona fide* CHI (Kaltenbach *et al*., 2018). The secondary structure of the active‐site cleft in type IV CHI is similar to *bona fide* CHI, while the key residues in the cleft are substituted. Thus, type IV CHI has neither catalytic activity nor fatty‐acid‐binding ability (Ngaki *et al*., 2012). Previous studies observed that type IV *CHI* exhibits a synchronous expression pattern with other genes involved in flavonoid biosynthesis (Yonekura‐Sakakibara *et al*., 2008). Mutations of type IV CHI in *Ipomoea* or *Arabidopsis* resulted in reduction of anthocyanins and PAs (Jiang *et al*., 2015; Morita *et al*., 2014). In addition, type IV CHI seems to physically interact with different enzymes in the flavonoid biosynthesis pathway in a species‐dependent or product‐dependent manner (Ban *et al*., 2018; Jiang *et al*., 2015). However, the molecular and biochemical basis of how type IV CHIs participate in and regulate flavonoid biosynthesis have not been well‐studied.

Numerous studies of the transcriptional regulation of flavonoid production have indicated that different sets of transcription factors (TFs) regulate the synthesis of specific class of flavonoids. The biosynthesis of flavonols has been reported to be regulated by a class of R2R3‐MYB factors targeting early biosynthetic genes (*EBGs*) and flavonol synthase (*FLS*) in *Arabidopsis thaliana* (Mehrtens *et al*., 2005) and fruits such as grape (*Vitis vinifera*) (Czemmel *et al*., 2009), apple (*Malus sieversii*) (Wang *et al*., 2017c) and orange (*Citrus sinensis*) (Liu *et al*., 2016). The production of anthocyanins and PAs is conservatively regulated by different MBW complexes, which are ternary complexes containing R2R3‐MYB TFs, basic helix‐loop‐helix (bHLH) TFs and WD‐repeat proteins (WDR) (Xu *et al*., 2014). A series of structural genes from *EBGs* to late biosynthetic genes [*LBGs*, e.g. *DFR*, *LDOX* (anthocyanin synthesis), *ANR* (PA synthesis)] can be the targets of these complexes (Xu *et al*., 2015). Since anthocyanins and PAs contribute to the fruit pigmentation and nutritional properties, the MBW complex has been characterized in many fruits, especially apple (Espley *et al*., 2007), grape (Hichri *et al*., 2010) and strawberry (*Fragaria × ananassa*) (Schaart *et al*., 2013). Although flavanones and flavones exhibit remarkable health‐promoting benefits (Li and Schluesener, 2017; Wenzel *et al*., 2000), unlike the extensive studies of the biosynthesis of flavonols, anthocyanins and PAs, little attention has been paid to the regulatory mechanism that determine their highly efficient accumulation in plants.

Citrus fruits are consumed worldwide and contain abundant bioactive flavonoids. Unlike most other fruits, which show a preference for the accumulation of flavonols and anthocyanins, the vast majority of the citrus species accumulate mainly flavanone glycosides and polymethoxylated flavones (PMFs) (Tripoli *et al*., 2007; Wang *et al*., 2017d) and are devoid of the ability to produce anthocyanins (Butelli *et al*., 2017). Although the flavanone glycosides and PMFs in citrus (CFLs) have attracted increasing attention due to their unique structures and potent pharmacological activities (Parhiz *et al*., 2015; Wang *et al*., 2019), the biosynthetic pathway and regulatory mechanism of CFL production are poorly understood. So far, there is no systematic characterization of the functions and enzymatic abilities of CHI and CHS in citrus. No type IV CHI protein in citrus has been identified, and little is known about whether it has a unique mechanism in regulating accumulation of CFLs. Despite being a potent enhancer of flavonoid production, the molecular basis underlying the transcriptional regulation of type IV CHI is enigmatic.

Ougan (*Citrus reticulata* cv. *Suavissima*) is a cultivated variety of the *Citrus* genus of the *Rutaceae* family, with abundant CFLs and no anthocyanin accumulation. Our previous report revealed that the PMFs extracted from Ougan have potent anti‐inflammatory effect (Wang *et al*., 2019). To further elucidate the regulatory mechanism governing CFLs biosynthesis in citrus and explore the role of type IV CHI in the pathway, we characterized the function of the citrus type IV CHI protein (CitCHIL1) and unravelled its transcriptional regulation. Results showed that CitCHIL1 played a critical role in the production of CFLs by forming a flavonoid metabolon. Three genes from the AP2/ERF superfamily were involved in the transcriptional regulation of *CitCHIL1*. Of these, CitRAV1 can interact with CitERF33 to form a transcription complex, which strongly promotes the expression of CitCHIL1 and the production of flavonoids. Our results not only deepen the understanding of type IV CHI function but also extend the knowledge of transcriptional regulation of flavanones and flavones.

## Results

### Identification and characterization of a type IV CHI gene from the *Citrus* genome

**Figure 1:**
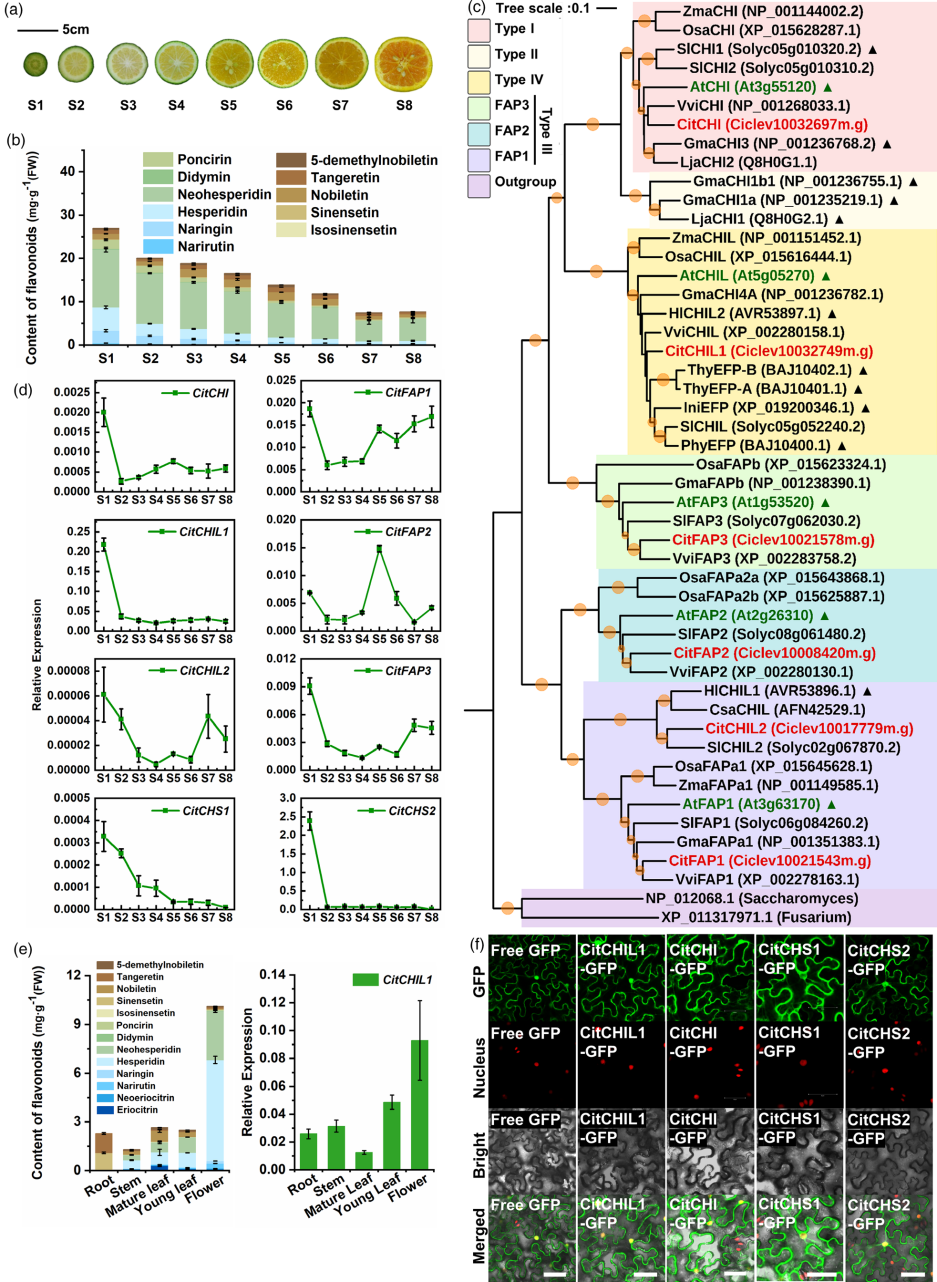
Flavonoid content in citrus and identification of *CitCHIL1* from the *Citrus* genome. (a) Ougan fruits were collected at eight developmental stages: S1 (30 days after flower blooming, DAFB), S2 (60 DAFB), S3 (80 DAFB), S4 (100 DAFB), S5 (120 DAFB), S6 (140 DAFB), S7 (170 DAFB), S8 (200 DAFB). (b) Measurement of CFLs in fruit peel in different developmental stages of Ougan. These results were the average of three biological replicates. Error bars denote SE of means of individual flavonoid content. (c) Phylogenetic analysis of CHI or CHI‐like (CHIL) proteins from citrus and other plants. The tree was constructed by MEGA‐X based on the multiple sequence alignment conducted by ClustalX and using the neighbour‐joining method rooted at Fungus CHI. The size of orange circles on the branches represents the bootstrap values based on 1000 bootstrap replicates (cut‐off was below 50%). The CHI/CHIL proteins of *Citrus* are shown in orange type and *Arabidopsis* members in green type. GenBank accession numbers are in parentheses. Functionally identified CHI/CHILs are marked with triangles. Species abbreviations: At, *Arabidopsis thaliana*; Cit, *Citrus clementina*; Csa, *Cannabis sativa*; Gma, *Glycine max*; Hl, *Humulus lupulus*; Ini, *Ipomoea nil*; Lja, *Lotus japonicus*; Osa, *Oryza sativa*; Phy, *Petunia hybrida*; Sl, *Solanum lycopersicum*; Thy, *Torenia hybrida*; Vvi, *Vitis vinifera*; Zma, *Zea mays*. (d) Quantitative RT‐PCR analysis of six *Citrus CHI* genes and two *Citrus CHS* genes at different developmental stages of Ougan peel. The transcript levels were expressed relative to citrus *β‐Actin* transcripts. The qRT‐PCR was repeated with three biological replicates, and error bars denote SE of means. (e) Measurement of CFLs and quantitative RT‐PCR analysis of *CitCHIL1* in different tissues of Ougan. These results are averages of three biological replicates. Error bars denote SE of means of individual flavonoid content. The transcript levels are expressed relative to citrus *β‐Actin* transcripts. (f) Subcellular localization of enzymes in the flavonoid biosynthetic pathway in transgenic *Nicotiana benthamiana* leaves (expressed together with nucleus‐located mCherry). The *A. tumefaciens* strain containing the empty eGFP vector was used as control. Bars = 50 μm.

A total of thirteen CFLs was identified in different tissues of Ougan, including eight flavanone glycosides and five PMFs (Figure S1a). Except for eriocitrin and neoeriocitrin that mostly accumulated in leaves and flowers, fruit peel mainly accumulated eleven kinds of CFLs (Figure S1b). Previous studies also showed that citrus peel contains much more flavonoids than pulp (Wang *et al*., 2017e), and thus, the peel was chosen for further study. During fruit development (Figure 1a), the flavonoid content in peel at developmental stage 1 (S1) was higher than that in other tissues and stages with a value of 26.97 mg/g fresh weight (FW), and continuously decreased from S1 to S7 (Figure 1b).

To identify the potential *CHI‐like* (*CHIL*) genes involved in CFLs production, we searched for the genes annotated to encode chalcone isomerase in the *Citrus clementina* genome database (https://www.citrusgenomedb.org/), and six putative *CHI‐fold* genes were identified. Phylogenetic analysis showed that these six proteins fell into three subfamilies excluding type II CHI subfamily (Figure 1c). A type IV CHI (designated as *CitCHIL1*) which showed the highest expression level of all *CHI‐fold* genes (about 140 times higher than *CitCHI* expression at S1) during fruit development was identified (Figure 1d), and its expression pattern showed high synchronicity with the accumulation of flavonoids (Figure S2). In addition, the *CitCHIL1* expression pattern was also coincident with that of *CitCHS1* (GenBank no. XM_006420545.2), *CitCHS2* (GenBank no. XM_006446839.2) and *CitCHI* (GenBank no. XM_006436077.2) (Moriguchi *et al*., 1999; Moriguchi *et al*., 2001) in peel during fruit development (Figure 1d), indicating that CitCHIL1 might be involved in the CFLs biosynthesis. In tissues other than fruit, flowers contained more flavonoids than roots, stems and leaves. Correspondingly, the expression level of CitCHIL1 in flowers was higher than that in other tissues (Figure 1e). Further, CitCHIL1 was colocalized with the encoded enzymes of other *EBGs* in the cytoplasm and nucleus (Figure 1f), providing a spatial basis for their participation in the same biosynthetic process.

*CitCHIL1* had an ORF of 630 bp, encoding a polypeptide of 209 amino acids. CitCHIL1 shared 68.42% and 78.47% amino acid sequence identity with AtCHIL (At5g05270) from *Arabidopsis* (Jiang *et al*., 2015) and HlCHIL2 (AVR53897.1) from *Humulus* (Ban *et al*., 2018), respectively.

### *In vitro* enzyme assays of recombinant CHIL1 protein

**Figure 2:**
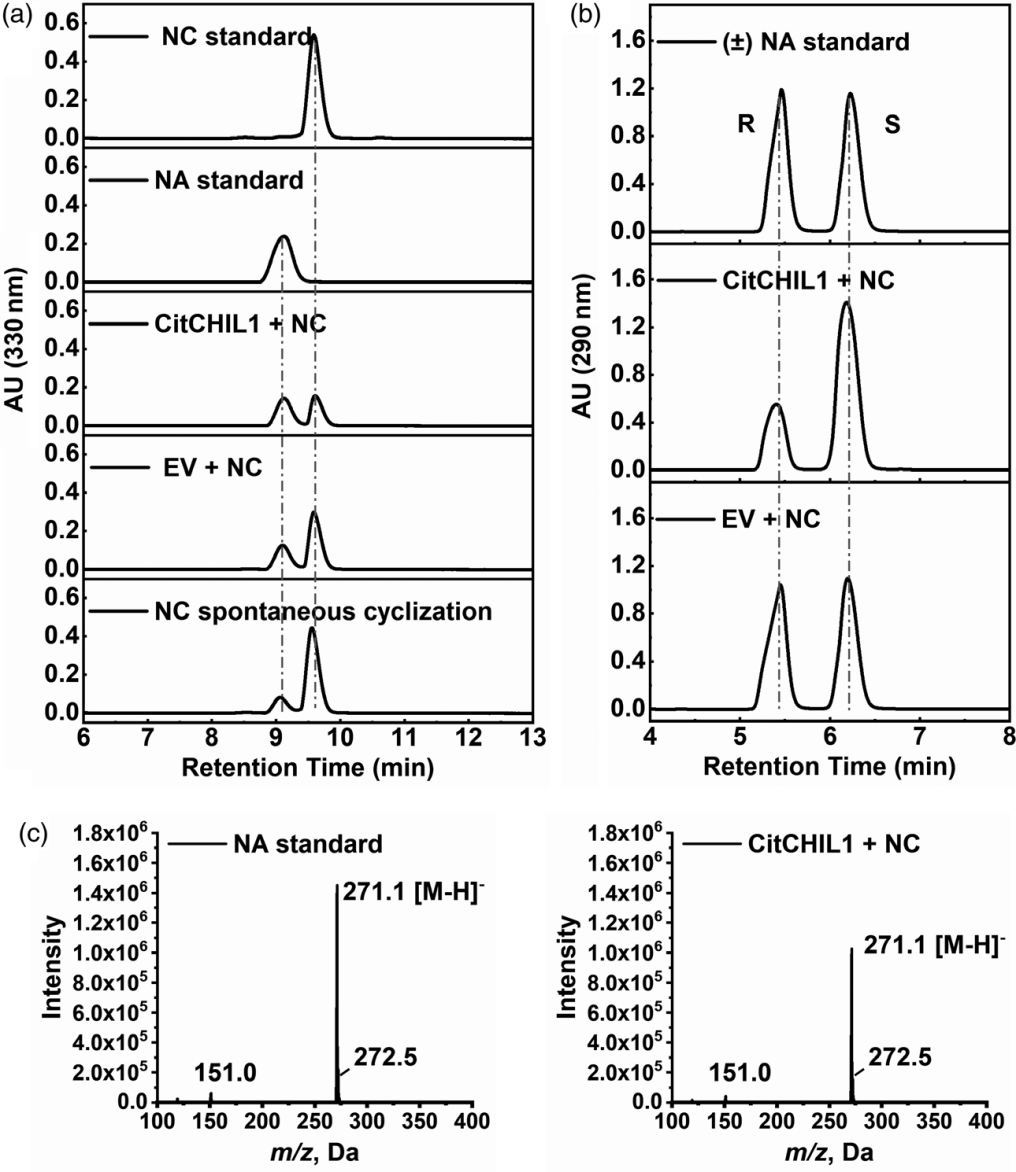
*In vitro* enzymatic assays of the recombinant CitCHIL1. (a) HPLC analyses of reaction products. The assays were conducted with naringenin chalcone as substrate, and 10 μm protein was added in each reaction. NC, naringenin chalcone; NA, naringenin; EV, proteins of empty pET32a vector. (b) The stereochemistry of recombinant CitCHIL1 catalysed product. HPLC profiles of the reaction products using a chiral separation column. (±) NA, racemic naringenin standard; R, (*2R*)‐naringenin; S, (*2S*)‐naringenin. (c) The mass spectra of naringenin standard and reaction products produced by incubation with CitCHIL1.

The structure‐based sequence alignment indicated that the catalytic residues of CitCHIL1 were all substituted, which suggested CitCHIL1 might be a noncatalytic CHI (Figure S3). However, surprisingly, although no activity of recombinant CitCHIL1 protein was detected with isoliquiritigenin (ILQ) as substrate (Figure S4a and S4c), we found that, compared with control protein (pET32a), high concentration of recombinant CitCHIL1 protein (10 μm) could help the conversion of naringenin chalcone to (*2S*)‐naringenin (Figure 2). When the content of CitCHIL1 protein in the reaction mixture was low (0.2 μm), there was no significant difference in product production compared with the control reaction (Figure S4b), whereas a detectable amount of product was found when the concentration of CitCHIL1 protein reached a high level (Figure 2a). The reaction product was further identified as naringenin (NA), with (*2S*)‐naringenin as the predominant stereoisomer, which was different from the racemic mixture of naringenin produced by the control reaction (Figure 2b‐c). These results revealed that CitCHIL1, as a type IV CHI, can assist with the cyclization of naringenin chalcone and has the stereospecificity to preferentially produce (*2S*)‐naringenin. However, this phenomenon could only be observed at high concentration of protein (>1 μm), and thus, it is difficult to determine its kinetic parameters.

### Physical interaction and regulatory effect of CHIL on CHS proteins

**Figure 3:**
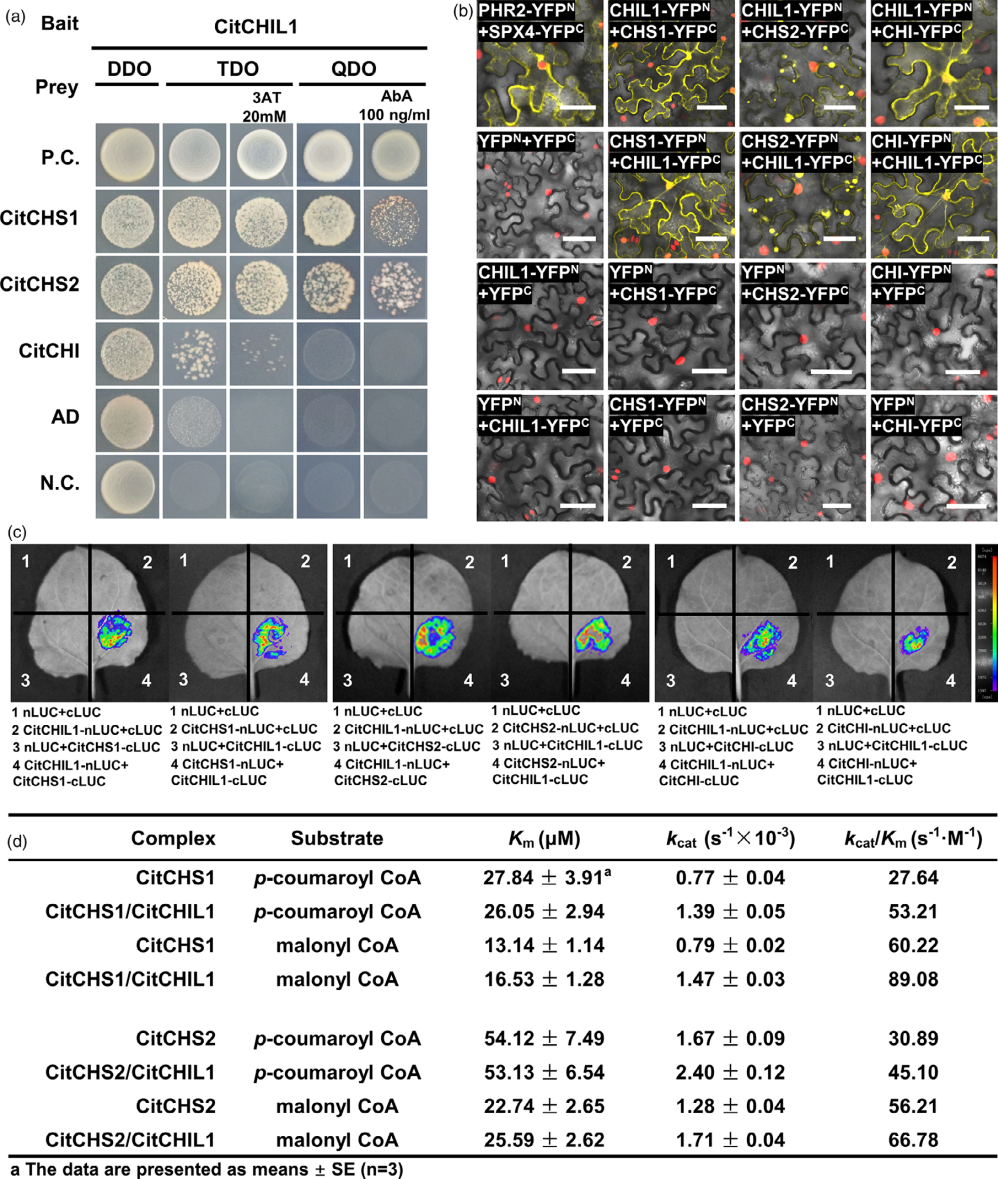
Physical interaction between CitCHIL1 and other *EBG*‐encoded proteins. (a) Yeast two‐hybrid analyses of the protein–protein interaction between CitCHIL1 and CitCHS1, CitCHIL1 and CitCHS2, CitCHIL1 and CitCHI. The TDO medium (SD‐Trp‐Leu‐His medium) and QDO medium (SD‐Trp‐Leu‐His‐Ade medium) were used as selective media. 3AT, 3‐amino‐1,2,4‐triazole; AbA, aureobasidin A; P.C., positive control, pGBKT7‐53 and pGADT7‐T constructs; AD, pGBKT7‐CitCHIL1 and pGADT7 constructs; N.C., negative control, pGBKT7 and pGADT7 constructs. (b) Bimolecular fluorescence complementation (BiFC) analyses of the protein–protein interactions between CitCHIL1 and CitCHS1, CitCHIL1 and CitCHS2, CitCHIL1 and CitCHI in transgenic *Nicotiana benthamiana* leaves (expressed together with nucleus‐located mCherry). The pairs of fusion proteins tested were CitCHIL1‐YFP^N^ + CitCHI‐YFP^C^, CitCHI‐YFP^N^ + CitCHIL1‐YFP^C^, CitCHIL1‐YFP^N^ + CitCHS1‐YFP^C^, CitCHS1‐YFP^N^ + CitCHIL1‐YFP^C^, CitCHIL1‐YFP^N^ + CitCHS2‐YFP^C^, and CitCHS2‐YFP^N^ + CitCHIL1‐YFP^C^. The pair PHR2‐YFP^N^ + SPX4‐YFP^C^was a positive control. The other combinations were negative controls. The yellow fluorescence visualized the interaction *in vivo*. Bars = 50 μm. (c) Firefly luciferase complementation imaging (LCI) analyses of the direct interaction between CitCHIL1 and CitCHS1, CitCHIL1 and CitCHS2, CitCHIL1 and CitCHI in *Nicotiana benthamiana* leaves. The luciferase images visualized the interaction *in vivo*. (d) Kinetic parameters of CitCHSs or CitCHIL1/CitCHS1 and CitCHIL1/CitCHS2 complexes with *p*‐coumaroyl‐CoA and malonyl‐CoA as substrates. For kinetic determination, malonyl‐CoA at a fixed concentration of 200 μm and *p*‐coumaroyl‐CoA at a series of concentrations (3–180 μm), or*p*‐coumaroyl‐CoA with a fixed concentration of 60 μm and malonyl‐CoA at a series of concentration (2–180 μm), were added to the standard CHS reactions. The *K*_m_ and *V*_max_ values were calculated by nonlinear regression curves fitted to the Michaelis–Menten equation using GraphPad Prism 8.0.

According to the results of yeast two‐hybrid (Y2H) assays, CitCHIL1 could interact with CitCHS1, CitCHS2 and CitCHI, respectively. The interaction of CitCHIL1 with CitCHI, however, was weaker than that with CHS proteins and the yeast containing CHIL1/CHI grew poorly on the selective medium (Figure 3a). Results of bimolecular fluorescence complementation (BiFC) analyses and firefly luciferase complementation imaging (LCI) analyses provided direct evidences *in vivo* to support this conclusion (Figure 3b‐c). The YFP signals and LUC signals only could be detected when the fusion proteins had the same subcellular localization and direct interaction. In LCI assays, the fluorescence intensity (cps) of CHIL/CHI complex was lower than that of CHIL/CHS complexes under the same imaging parameters, supporting the results of Y2H assays.

To further examine the differences in the catalytic efficiency of CitCHIL1 and compare it with the other two CHS proteins, we used *p*‐coumaroyl‐CoA and malonyl‐CoA as substrates to study the chalcone synthase reaction *in vitro* with or without CitCHIL1 as auxiliary protein. The substrate affinity (*K*_m_ value) and catalytic constant (*k*_cat_ value) of the CitCHS1 and CitCHS2 differed. CitCHS1, with lower *K*_m_ values, showed a higher affinity for substrates, while CitCHS2 exhibited a higher conversion rate of substrates (Figure 3d and Figure S5), resulting in a similar catalytic efficiency (*K*_m_/*k*_cat_ value) of the two enzymes. The presence of CitCHIL1 had little effect on the *K*_m_ values of the two enzymes, but improved the *k*_cat_ values to varying degrees. The *k*_cat_ value of CitCHS1 increased by 1.81‐fold for *p*‐coumaroyl‐CoA and 1.86‐fold for malonyl‐CoA, respectively. These values were higher than the value for CitCHS2, which increased by 1.43‐fold for *p*‐coumaroyl‐CoA and 1.33‐fold for malonyl‐CoA, respectively (Figure 3d and Figure S5).

### Overexpression and RNA interference‐induced silencing of *CitCHIL1 in planta*

**Figure 4:**
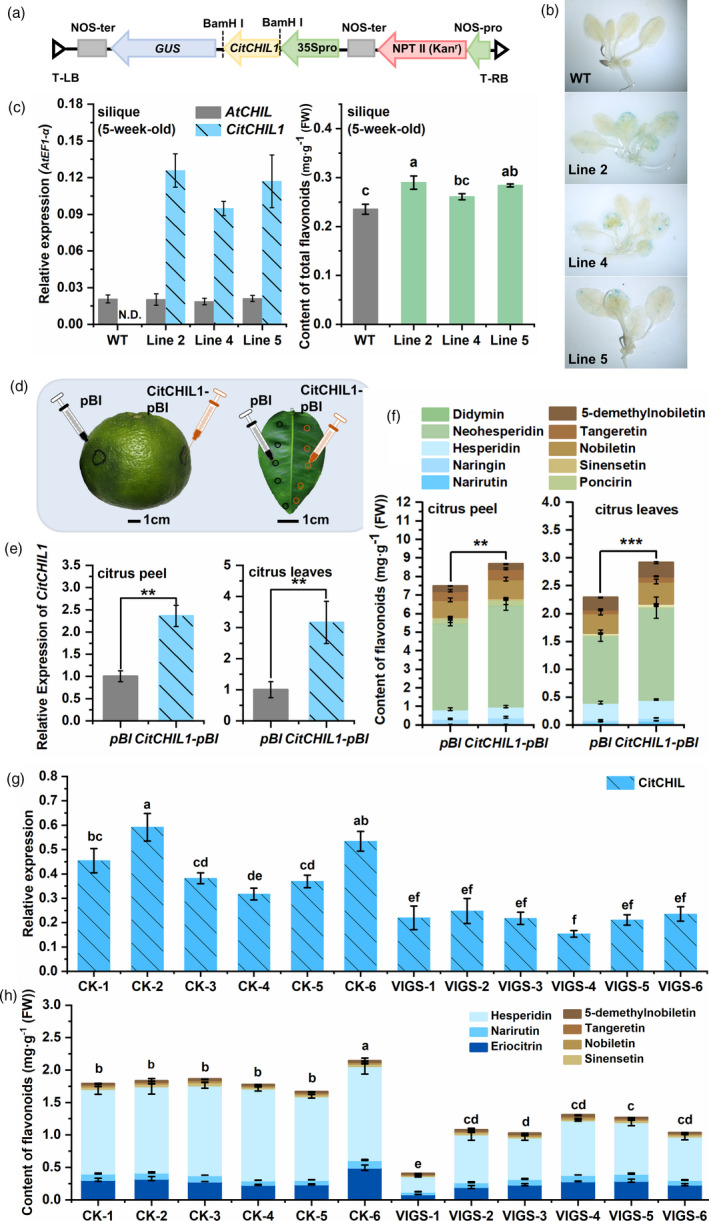
Overexpression and RNA interference‐induced silencing of *CitCHIL1 in planta*. (a) Schematic map of the CitCHIL1‐pBI121 construct. (b) GUS (β‐glucuronidase) staining of wild‐type (WT) and transgenic *Arabidopsis* 3‐week‐old seedlings. (c) Quantitative RT‐PCR analysis of *CitCHIL1* and *AtCHIL* and determination of total flavonoid content in WT and transgenic *Arabidopsis*. The transcript levels are expressed relative to AtEF1‐α transcripts. The content of total flavonoids in WT plants was 0.235 mg/g FW. Values are means ± SE (*n* = 3). Significant differences were determined by one‐way ANOVA test: *, *P* < 0.05; **, *P* < 0.01; ***, *P* < 0.001. (d) Schematic diagram for injection of citrus fruit and leaves. (e) Relative expression of *CitCHIL1* in CitCHIL1‐pBI overexpressing fruit or leaves 5 d after injection. (f) Measurements of CFLs in citrus peel and leaves 5 d after transient overexpression. In e and f, pBI, injection of *A. tumefaciens* strain (EHA105) with empty pBI121 vector; CitCHIL1‐pBI, injection of *A. tumefaciens* strain (EHA105) with pBI121‐*CitCHIL1* construct. Error bars indicate SE from eight replicates. Statistical significance was determined by one‐tailed paired *t*‐test: **, *P* < 0.01; ***, *P* < 0.001. (g) Relative expression of *CitCHIL1* in virus‐induced *CitCHIL1* silencing citrus seedlings. (h) Measurements of CFLs in virus‐induced *CitCHIL1* silencing citrus seedlings. In g and h, CK, empty TRV2 vector control plants; VIGS, virus‐induced *CitCHIL1* gene silencing plants. Error bars indicated SE from three technical repetitions. Statistical significance was determined by one‐way ANOVA and Duncan’s test.

LCI assays showed that there was physical interaction between CitCHIL1 and AtCHS (At5g13930) (Figure S6a), consistent with the previous research of HlCHIL2 in *Humulus* (Ban *et al*., 2018), indicating that CitCHIL1 might also improve the catalytic activity of AtCHS. Thus, the function of CitCHIL1 was validated *in vivo* in transgenic *Arabidopsis* plants that overexpressed *CitCHIL1* in the wild‐type (WT) *A. thaliana* (Col0) (Figure 4a‐c). Histochemical staining of β‐glucuronidase (GUS staining) and RT‐qPCR analyses showed that *CitCHIL1* was stably expressed in the transgenic lines (Figure 4b‐c). In *Arabidopsis*, the flavonoids were accumulated mostly in the inflorescences and siliques (Stracke *et al*., 2010) and we thus determined the total flavonoids in siliques of 5‐week‐old WT and transgenic plants (T_3_). After hydrolysis, the main flavonoid aglycones in Arabidopsis were quercetin and kaempferol, so we calculated the content of these two compounds as total flavonoids (Figure S6b). Compared with WT plants, the content of total flavonoids in three CitCHIL1‐OE lines significantly increased by 1.11‐ to 1.23‐fold (Figure 4c), whereas the transcript abundance of endogenous AtCHIL in transgenic lines was unchanged.

Homologous overexpression and RNAi manipulations were carried out to confirm the flavonoid accumulation‐enhancing function of CitCHIL1 in citrus, using Ougan or Jincheng (*Citrus sinensis* (L.) Osbeck) as materials (Figure 4d‐h). Compared with the empty vector control (pBI), overexpression of *CitCHIL1* in Ougan peel significantly increased the total CFLs content by 1.16‐fold (*P* < 0.05), and overexpression of *CitCHIL1* in Ougan leaves increased the total CFLs content by 1.27‐fold compared to the control (*P* < 0.05; Figure 4f). Virus‐induced gene silencing (VIGS) of *CitCHIL1* was performed using Jincheng germinated seedlings (Figure 4g‐h). Compared with the control plants (transformed with empty vector TRV2), the expression level of *CitCHIL1* decreased to various degrees in the six VIGS‐positive plants (Figure 4g). The reduced gene expression of the positive plants resulted in a significant decrease in the total flavonoid content (Figure 4h). These results offered direct evidence that *CitCHIL1* is involved in the early biosynthesis of flavonoid *in planta* and effectively promotes the flavonoid production.

### Screening of potential transcription factors activating CitCHIL1 transcription

**Figure 5:**
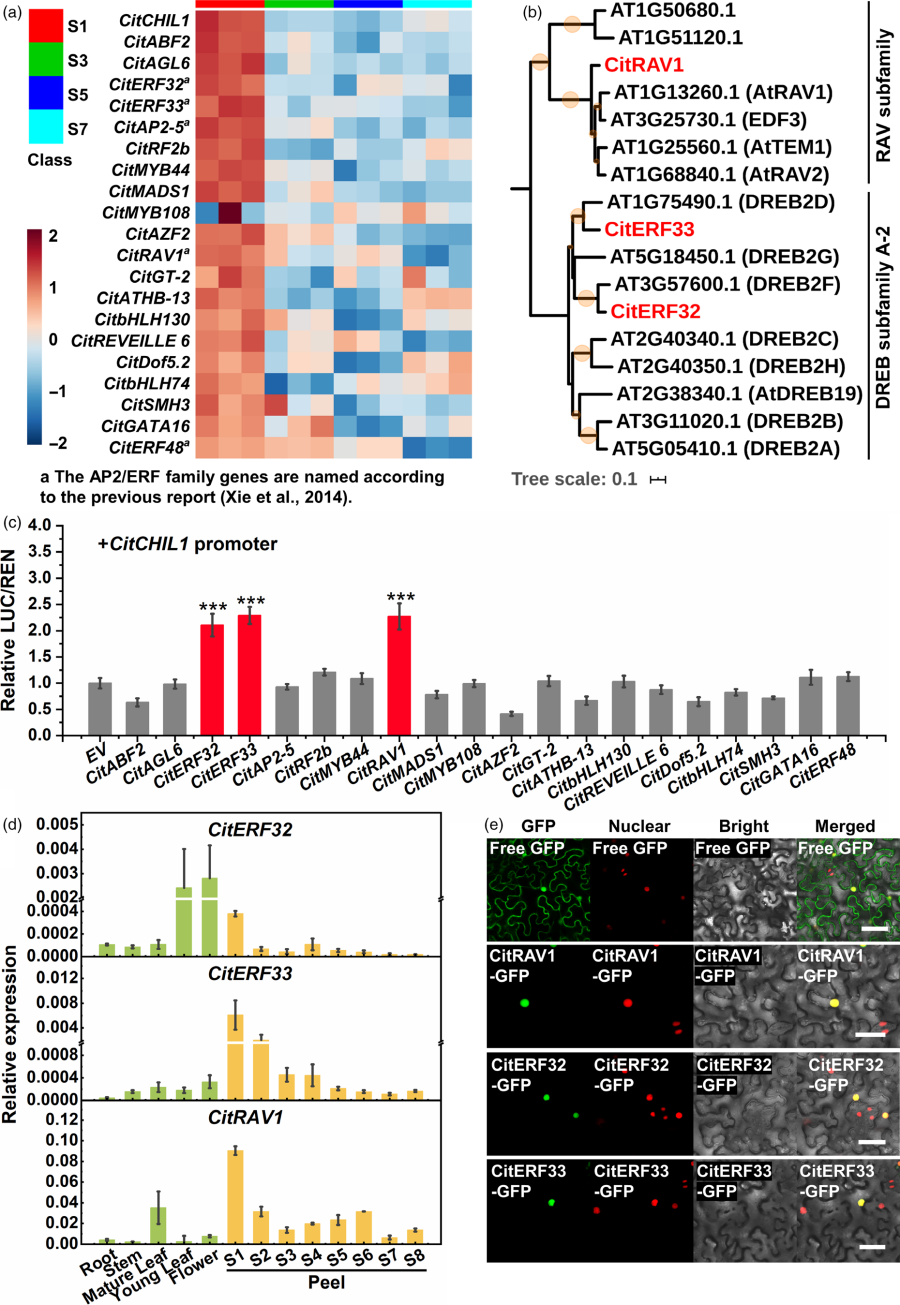
Identification of TFs activating *CitCHIL1* promoter. (a) Heatmap of expression profiles of potential TFs in citrus peel during fruit development. The RNA‐seq was conducted with three biological replicates. Transcript abundance of TFs was represented by lg (FPKM value). The heatmap was constructed with online software MetaboAnalyst v.4.0. (b) Phylogenetic analysis of CitERF32, CitERF33, CitRAV1 and members of *Arabidopsis* DREB A‐2 subfamily and RAV subfamily. The tree was constructed using the neighbour‐joining method. At, *Arabidopsis thaliana*. The size of orange circles on the branches represents the bootstrap values based on 1000 bootstrap replicates (lower than 50% was cut‐off). The TFs of *Citrus* are shown in red type. Names of the genes from *Arabidopsis* are in parentheses. (c) Regulatory effects of potential TFs on the promoter of *CitCHIL1*. Firefly luciferase/Renilla luciferase (LUC/REN) values obtained with the empty pGreenII‐SK vector and the *CitCHIL1* promoter were set as 1, and SE values were calculated from six replicates. Each TFs–promoter interaction was confirmed by three independent experiments, and each experimental point had at least six biological replicates. Statistical significance was determined by one‐way ANOVA and LSD test: ***, *P* < 0.001. (d) Quantitative RT‐PCR analysis of *CitERF32*, *CitERF33* and *CitRAV1* in different tissues and developmental stages of citrus. The transcript levels are expressed relative to citrus *β‐Actin* transcripts. Values are means ± SE (*n* = 3). (e) Subcellular localization of CitERF32, CitERF33 and CitRAV1 in transgenic *Nicotiana benthamiana* leaves (expressed with nucleus‐located mCherry). Empty eGFP vector used as control was the same to the control of Figure 1f. NLS‐mCherry, nucleus‐localized signal. Bars = 50 μm.

A 2074‐bp fragment of the promotor region of *CitCHIL1* was cloned upstream of the ATG codon. A total of 14 TFs were screened by yeast one‐hybrid (Y1H) library screening with *CitCHIL1* promoter as bait (Table S1). Unfortunately, none of these TFs had binding activity or any activation effect in the subsequent verification process (Figure S7). We carried out online TF‐binding site prediction using the website PlantTFDB (Tian *et al*., 2019). This identified a total of 148 TFs, which fell into 27 families, that were indicated as possibly being able to bind to the promoter (Table S2). The expression patterns of these TFs and *CitCHIL1* in Ougan were compared using the FPKM values (mean value of three replicate at each stage) generated from transcriptome sequencing (RNA‐seq) at the four developmental stages (S1, S3, S5, S7). TFs with positive regulatory effects were carefully analysed, and twenty candidate TFs with Pearson’s correlation coefficient ≥ 0.7 were selected (Figure 5a). These TFs belonged to 13 families, including five members of the AP2/ERF family.

### Activation effect of three AP2/ERF superfamily TFs on the *CitCHIL1* promoter

Three TFs, that is CitERF32, CitERF33 and CitRAV1 [named according to a previous study (Xie *et al*., 2014)], were verified to be able to activate the *CitCHIL1* promoter by dual‐luciferase assays in tobacco leaves (threshold value was set as 2), with increment of 2.1‐fold, 2.3‐fold and 2.3‐fold, respectively (Figure 5b‐c). Interestingly, all of these are members of the AP2/ERF superfamily. Phylogenetic analysis showed that CitERF32 and CitERF33 were homologous to DREB2D and DREB2F in *Arabidopsis* (Figure 5b). Although both CitERF32 and CitERF33 are DREB subfamily A‐2 members, they shared low similarity at the amino acid level except for the APETALA2 (AP2) domain. RT‐PCR analyses of transcript levels of these three TFs confirmed the results of RNA‐seq and provided information about spatial expression (Figure 5d). *CitRAV1* was mainly expressed in fruit and mature leaves, with a slight expression rise from S2 to S6 in fruit. *CitERF33* exhibited similar tissue specificity with *CitCHIL1,* and the transcript abundance of *CitERF32* was higher in flower and young leaf than in fruit. Subcellular localization analyses revealed that all three of these TFs were localized in the nucleus (Figure 5e).

### CitERF32 and CitERF33 directly bind to the *CitCHIL1* promoter

**Figure 6:**
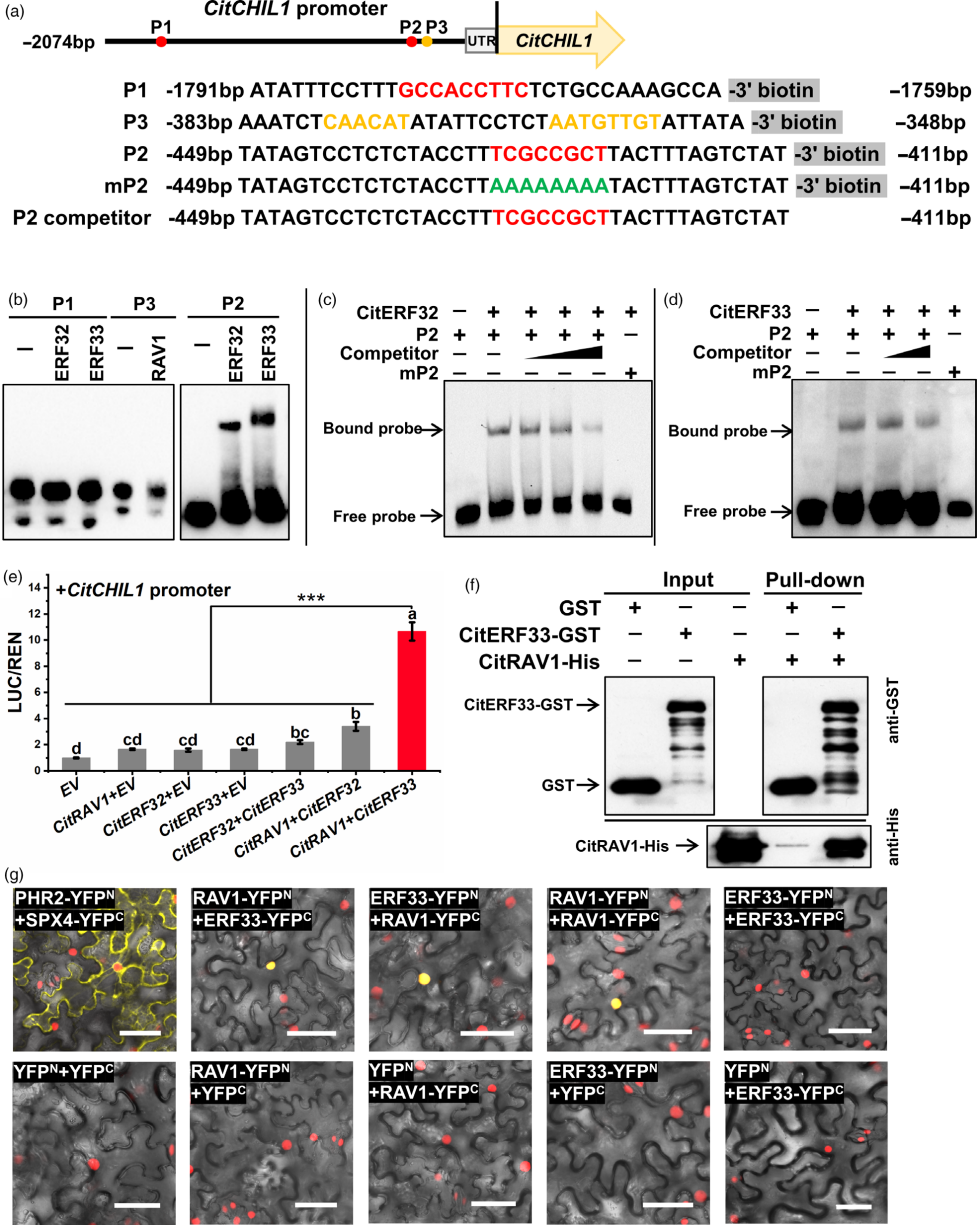
Analyses of direct DNA–protein interaction between *CitCHIL1* promoter and TFs and protein–protein interaction between TFs. (a) Schematic representations of *CitCHIL1* promoters and the probes used for the electrophoretic mobility shift assay (EMSA). DREB‐binding sites are represented in red, and RAV core sequence is represented in yellow. The mutated bases are indicated in green. P1 and P2, probe 1 and probe 2, predicted binding sites of CitERF32 and CitERF33; P3, probe 3, predicted RAV1‐binding motif. P2 competitor, probe 2 without 3' biotin modification; mP2, mutant probe 2. (b) Preliminary EMSA. (c‐d) EMSAs of probe 2 with CitERF32 (c) and CitERF33 and (d) DNA‐binding domain protein. The competition experiments were conducted with 200, 500 or 1000 molar excess of unlabelled probes. In c‐d, water was added in place of TF proteins as a control. (e) Combined activation effects of CitERF33/CitRAV1 on the *CitCHIL1* promoter. LUC/REN values obtained with the empty pGreenII‐SK vector and the *CitCHIL1* promoter were set as 1, and error bars represented SE values calculated from six replicates. Each TFs–promoter interaction was confirmed by three independent experiments, and each experimental point had at least six biological replicates. Different letters above the bars indicated significant differences (*P* < 0.05) obtained by one‐way ANOVA and LSD test. Asterisks denote *t*‐test significance: ***,*P* < 0.001. (f) GST pull‐down assays of the protein–protein interaction between CitRAV1 and CitERF33 *in vitro*. The protein of empty GST vector was used as the control. (g) Bimolecular fluorescence complementation (BiFC) analyses of the protein–protein interaction between CitRAV1 and CitERF33 in transgenic *Nicotiana benthamiana* leaves (expressed with nucleus‐located mCherry). The pairs of fusion proteins tested were CitERF33‐YFP^N^ + CitRAV1‐YFP^C^, CitRAV1‐YFP^N^ + CitERF33‐YFP^C^, CitERF33‐YFP^N^ + CitERF33‐YFP^C^, CitRAV1‐YFP^N^ + CitRAV1‐ YFP^C^. The pair PHR2‐YFP^N^ + SPX4‐YFP^C^ was a positive control. The other combinations were negative controls. The yellow fluorescence visualized the interaction *in vivo*. Bars = 50 μm.

The dehydration‐responsive element (DRE) with a core sequence (A/G)CCGAC is a specific binding motif for many DREB proteins reported, including AtDREB1A and AtDREB2A (Liu *et al*., 1998; Sakuma *et al*., 2002). However, we did not find the (A/G)CCGAC motif in the *CitCHIL1* promoter. CitERF32 and CitERF33 were predicted by PlantTFDB to bind to GCCACCTTC motif (marked as P1) or CGCCGC motif (marked as P2) in *CitCHIL1* promoter, which are very similar to the binding sites of their homologous genes *AtDREB2G* and *AtDREB2D* recorded in Plant Cistrome Database (O'Malley *et al*., 2016). The RAV subfamily proteins contain two DNA‐binding motifs: an AP2 domain and a B3‐like domain, which specifically binds to gCaACA(g/t)(g/t) motif (simplified as CAACA) and caCCTG(a/g) motif, respectively (Kagaya *et al*., 1999). Either the AP2 domain or the B3‐like domain is capable of binding to DNA sequences on its own. According to the prediction by PlantTFDB, the *CitCHIL1* promoter sequence has two adjacent CAACA‐binding sites marked as P3 (Figure 6a) and no caCCTG(a/g) motif was found in the promoter. Results of dual‐luciferase assays with the truncated promoters suggested that the three TFs only bound to the elements located between −500 and −200 bp of the promoter (Figure S8). Similarly, the results of EMSA indicated that both CitERF32 and CitERF33 were able to bind to probe P2 rather than to probe P1 (Figure 6b). The competitive binding assays with unlabelled probe validated the specificity of the protein–DNA interaction (Figure 6c and d). Mutation of the P2 core sites CGCCGC completely abolished the binding, indicating the specific binding activity between two DREB proteins and the probe P2. Unexpectedly, by contrast, no shifted band appeared in the assay of CitRAV1 + probe P3, suggesting that CitRAV1 may not directly bind with the *CitCHIL1* promoter (Figure 6b).

### CitRAV1 interacts with CitERF33 to form a transcriptional complex and enhance the transcription effects

Considering the contradiction between the results of EMSA and results of dual‐luciferase assays with CitRAV1, we suspected that CitRAV1 may activate the transcription of *CitCHIL1* in an indirect way *in vivo*, for example by forming a transcription complex with other proteins that could bind directly to the *CitCHIL1* promoter. Therefore, we studied the potential interactions between CitRAV1 and CitERF32 or CitERF33. Dual‐luciferase assays with two TF proteins indicated that the combination of CitERF33 and CitRAV1 generated a synergistic 10.6‐fold activation effect on the *CitCHIL1* promoter, comparing to the 1.6‐fold activation of CitERF33/EV or CitRAV1/EV individually (Figure 6e). No significant synergistic activation effects were observed with the combination of CitERF32/CitERF33 or CitRAV1/CitERF32 (set threshold value as 4). Pull‐down assays and BiFC assays verified the interaction between CitRAV1 and CitERF33 *in vitro* and *in vivo*, respectively. In pull‐down assays (Figure 6f and Figure S9), the CitRAV1‐His fusion protein was pulled down by the CitERF33‐GST fusion protein and detected by the His antibody. In BiFC assay, the combinations of CitRAV1‐YFP^N^/CitERF33‐YFP^C^, CitRAV1‐YFP^C^/CitERF33‐YFP^N^ and CitRAV1‐YFP^N^/CitRAV1‐YFP^C^ exhibited fluorescence signals in the nucleus (Figure 6g), suggesting that CitRAV1 was able to form homodimers or multimers, and also interact with CitERF33 to form a transcription complex, which showed strong transcription activation on the *CitCHIL1* promoter.

### Effects of overexpressing TFs in citrus leaves

**Figure 7:**
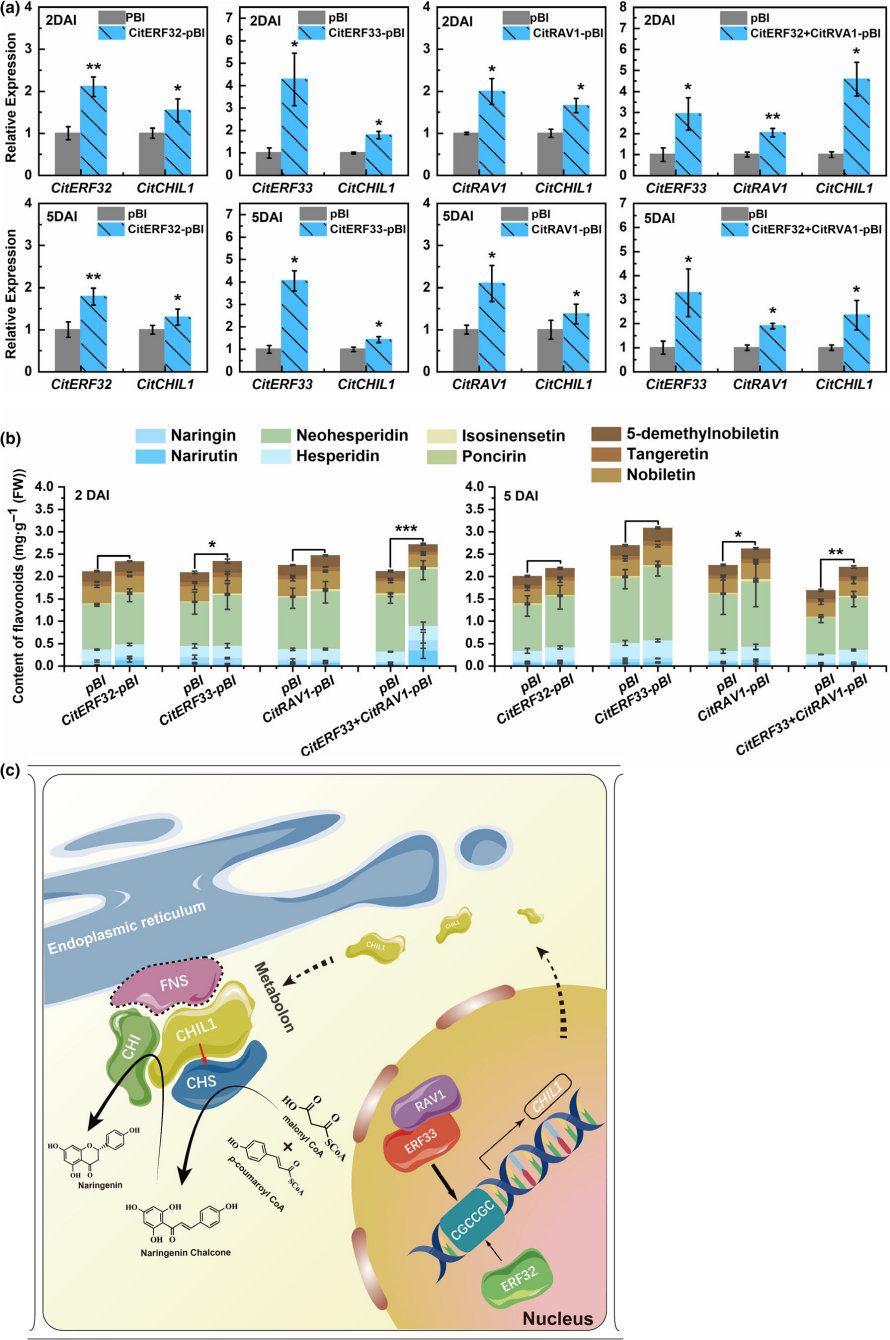
Transient overexpression of three TFs in citrus leaves and a proposed model of TFs and CitCHIL1 function in flavonoid production *in vivo*. (a) Gene expression of *TFs* and endogenous *CitCHIL1* in citrus leaves 2 days or 5 days after injection for transient expression. The transcript level of CitCHIL1 on the control side of leaves [injection of *A. tumefaciens* strain (EHA105) with the empty pBI121 vector] was set as 1 in each group. DAI, days after injection. SE values were calculated from at least four biological replicates. Statistical significance was determined by one‐tailed paired *t*‐test (*, *P* < 0.05; **, *P* < 0.01; ***, *P* < 0.001). (b) The measurement of metabolites in citrus leaves 2 days or 5 days after injection. DAI, days after injection. SE values were calculated from at least four biological replicates. Statistical significance was determined by one‐tailed paired *t*‐test (*,*P* < 0.05; **, *P* < 0.01; ***, *P* < 0.001). (c) CitCHIL1 interacts with CHS and CHI to function as a component of the flavonoid metabolon, and CitCHIL1 assists the cyclization of naringenin chalcone, thus ensuring the efficient influx of substrates to the flavonoid pathway and improving the efficiency of CHS. CitERF32 and CitERF33 bind to the *CitCHIL1* promoter, respectively, and activate its expression. CitRAV1 interacts with CitERF33 in the nucleus to form a transcription complex, thereby strongly activating the transcription of *CitCHIL1* and promoting the accumulation of flavonoids. Dotted‐line, unreported protein. The graphic characters neither represent the real structure of the proteins nor represent a real stoichiometric relationship.

Homologous transient overexpression was carried out to verify the function of the individual TFs and the transcription complex in flavonoid biosynthesis *in vivo*, using Ougan leaves as material due to their low background values of flavonoids and low *CitCHIL1* transcript level (Figure 7a‐b). Compared with the control, overexpressing single TFs significantly increased the transcript level of *CitCHIL1* to varying degrees at 2 day or 5 day after infiltration. Coexpression of *CitRAV1/CitERF33* on the other hand increased *CitCHIL1* expression by 4.59‐fold (2 day after infiltration) or 2.35‐fold (5 day after infiltration), which was significantly higher than that obtained with overexpression of either *CitRAV1* or *CitERF33* (Figure 7a). Overexpression of *CitRAV1/CitERF33* together resulted in a 28% (2 day after infiltration) and 31% (5 day after infiltration) increment in total flavonoid content in citrus leaves. In the leaves infiltrated with individual TFs, the total flavonoid contents were also higher than control in varying degrees (Figure 7b). These results provided direct evidences that all three TFs tested function as positive regulators of flavonoid biosynthesis by activating *CitCHIL1* transcription, while the CitERF33/CitRAV1 transcription complex further enhanced the efficiency of flavonoid biosynthesis.

## Discussion

### CitCHIL1 can assist the orientational cyclization of naringenin chalcone

Prior studies have considered that type IV CHI proteins are the ancestors of the *bona fide* CHI but have no catalytic activity due to lack of key active residues (Ngaki *et al*., 2012), and CHILs from many species, including *Glycine max* (Ralston *et al*., 2005) and *A. thaliana* (Ngaki *et al*., 2012), have been shown to be noncatalytic. Notably, the *in vitro* enzyme assays employed in those studies often used low concentration of proteins. Here, we found that high concentration of CitCHIL1 could promote the cyclization rate of naringenin chalcone and increase the formation of the (*2S*)‐isomer product. Although this process is faster than spontaneous cyclization, it does not achieve the rate of enzymatic catalysis. Since some members of type III CHIs can bind to chalcone (Ban *et al*., 2018), we speculate that the substrate can interact with enzyme catalytic cavity and selectively and slowly cyclize into the S‐isomer in this cavity. This would explain why high concentrations of the enzyme can amplify this weak reaction to a noticeable level. Determining the protein crystal structure to visualize the molecular docking might verify this hypothesis. These results demonstrated the previous conjecture that the ancestors of CHI may have a promiscuous and inefficient enzyme activity based on the evolutionary trajectory and structural analysis of key amino acids (Kaltenbach *et al*., 2018), and also provide a possible explanation of how bryophyte species such as *Physcomitrella patens* could accumulate flavonoids in the absence of *bona fide* CHI (Cheng *et al*., 2018; Koduri *et al*., 2010).

### CitCHIL1 is a component of a CFL biosynthetic metabolon and accelerates the influx of substrates to CHS

A metabolon is a structural–functional complex of interacting enzymes involved in a metabolic pathway, allowing for the effective control of metabolic flux. The components of a flavonoid metabolon seem to be product‐dependent and species‐dependent. In *Antirrhinum majus* and *Torenia hybrida*, CHI plays a critical role in forming a flavonoid metabolon by interacting with membrane‐localized proteins and DFR (Fujino *et al*., 2018). Since *bona fide* CHI evolved from type IV CHI, it can be inferred that the function of *bona fide* CHI interactions with other enzymes in the metabolon was inherited from type IV CHI (Ban *et al*., 2018). Unlike *Arabidopsis* (Jiang *et al*., 2015), the transcript level of *CitCHIL1* was much higher than that of *CitCHI* (Figure 1d), suggesting that type IV CHI might function differently in different species. Taken together, we propose a hypothetical model of flavonoid metabolon which is citrus‐specific (Figure 7c): a protein with membrane localization [e.g. flavone synthase (FNS)] is firstly tethered to the endoplasmic reticulum (ER), and then, CitCHIL1 physically interacts with FNS (or certain flavonoid hydroxylases), CHI and CHS, respectively, and ensures a high‐efficiency metabolic flux for flavonoid production.

The mechanism whereby CHIL regulates the catalytic efficiency of CHS is still unknown. HlCHIL2 in hops can increase the catalytic efficiency of CHS‐H1 and PT1L *in vitro*, and change their *K*_m_ values, leading to assumption that CHIL may remodel the active‐site cavity of CHS (Ban *et al*., 2018). However, our data show that interaction with CitCHIL1 does not significantly change the substrate affinity of two CHSs and CitCHIL1 but can promote the catalytic efficiency of different CHSs to an approximately similar extent *in vitro*. Furthermore, *in vivo* assays of recombinant proteins showed that CitCHIL1 could promote the orientational isomerization of naringenin chalcone. Therefore, we speculate that CHS‐CitCHIL1 forms a protein complex which may accelerate the transformation and transfer of naringenin chalcone, thus alleviating the product feedback inhibition of CHS and improving the efficiency of naringenin chalcone production. This could also explain why CHIL could rectify the promiscuous specificity of CHS (Waki *et al*., 2020). Previous studies have mostly verified the function of CHIL *in vivo* by knockdown or mutation (Jiang *et al*., 2015; Morita *et al*., 2014). Overexpression results in this study provide another perspective and strongly confirm the role of CHIL as an enhancer of flavonoid production in *Citrus* and *Arabidopsis*. Further, the result of CitCHIL1 in citrus extends the understanding of the function of type IV CHI from herbaceous plants to perennial trees.

### In citrus, CFL production is likely to be regulated by AP2/ERFs

Multiple lines of evidence indicate that the regulatory mechanism governing flavanone and flavone production is different from that of flavonols and anthocyanins, although all of them are synthesized through similar *EBGs*. Since most of the citrus cultivars are unable to produce anthocyanins, citrus fruit is the ideal material for studying the biosynthetic mechanisms of flavanones and flavones in fruit. Of the 148 TFs predicted by PlantTFDB, *ruby1* was not expressed and most other MYB factors exhibited expression patterns uncorrelated or negatively correlated with flavonoid accumulation during fruit development (Table S2). These results indicated that MYB factors might not play a key role in regulating CFL production.

In contrast, our results revealed that accumulation of transcripts of three AP2/ERF superfamily TFs was correlated with CFL synthesis and could activated the *CitCHIL1* promoter (Figure 5). It is well known that AP2/ERF family TFs participate in many aspects of plant physiological processes and play crucial roles in regulating fruit quality (Li *et al*., 2017; Wang *et al*., 2017b; Zeng *et al*., 2015). However, AP2/ERF TFs have rarely been reported previously to directly regulate the structural genes of flavonoid biosynthesis.

Both CitERF32 and CitERF33 are members of the DREB‐2A subfamily. The function of their homologous genes in *Arabidopsis*, DREB2D and DREB2F, is not clear. However, many other members of the DREB subfamily have been shown to respond to abiotic stress [AtDREB1 and AtDREB2 (Liu *et al*., 1998); OsDREBs (Dubouzet *et al*., 2003); TaDREB2 and TaDREB3 (Morran *et al*., 2011; Yang *et al*., 2019)]. Whether abiotic stress, such as low‐temperature or drought, could affect the biosynthesis of CFLs by inducing *CitERF32* or *CitERF33* transcription remains to be tested by further investigation. Five DREB TFs have been reported in citrus (Xie *et al*., 2014), and further studies are required to see whether CitERF32 and CitERF33 execute different functions in different stress responses.

### A DREB‐RAV transcription complex within the AP2/ERF family strongly promotes *CitCHIL1* expression and CFL biosynthesis

Many TFs play important roles in the biosynthesis of flavonoids by forming transcription complexes with other TFs, such as MBW complexes (Xu *et al*., 2015). There have, however, been few reports of interaction between members of the AP2/ERF family. Our results revealed that CitERF33 can interact with CitRAV1 to form a transcriptional complex, which explains the previous observation that the targets of MaDREB2 include many other TFs including ERFs in banana (*Musa acuminata*) (Kuang *et al*., 2017), indicating that DREB2 proteins can participate in regulatory cascade networks and affect physiological processes.

RAV1 in *Arabidopsis* is a versatile negative regulator of plant growth, drought resistance and salt resistance, and a positive regulator of leaf senescence (Fu *et al*., 2014; Hu *et al*., 2004; Woo *et al*., 2010). *RAV1* is also sensitive to a variety of plant hormones (Feng *et al*., 2014; Zhao *et al*., 2019). In this study, the expression pattern of CitRAV1 transcript accumulation suggested it may also be involved in the process of fruit ripening as well as leaf senescence, which was consistent with previous observations (Woo *et al*., 2010). Recently, RAV1 from strawberry was reported to regulate anthocyanin accumulation by directly activating transcription of FaMYB10 and other genes, excluding CHI (Zhang *et al*., 2020). In the present study, we showed that although CitRAV1 cannot directly bind to the promoter of *CitCHIL1* either, it can form homologous dimers or oligomers and combine with CitERF33 to form transcriptional complexes (Figure 6), which has not been previously described. Anthocyanin formation is often regulated by MYBs and other TFs (Butelli *et al*., 2017) but this does not occur in Ougan, which provides a unique opportunity to study the mechanism of RAV1 in the regulation of flavanones and flavones. In regulating plant resistance, DREB and RAV1 showed opposing effects, indicating that the biosynthesis of CFLs might involve a complex network of interacting factors. These findings are important for generating plants with various levels of flavonoids and also indicate the importance of further studies on the relationship between CFLs and stress response.

In conclusion, we found that CitCHIL1 promotes the synthesis of flavanones and flavones in citrus by participating in flavonoid metabolon and thus improving the efficiency of CHS. The *CitCHIL*1 gene is transcriptionally activated by three AP2/ERF TFs, and a DREB‐RAV complex significantly enhanced transcription of *CitCHIL1* and accumulation of citrus flavonoids (Figure 7c). These results will be of great significance for further understanding and manipulating the mechanism of flavonoid synthesis in plants.

## Experimental procedures

### Plant materials and chemicals

Ougan (*Citrus reticulata cv. Suavissima*) tissues including roots, stems, leaves, flowers and fruits were obtained from Wenzhou, China. Fruits were harvested at eight different developmental stages, S1 (30 days after flower blooming, DAFB), S2 (60 DAFB), S3 (80 DAFB), S4 (100 DAFB), S5 (120 DAFB), S6 (140 DAFB), S7 (170 DAFB), S8 (200 DAFB), and the peels were separated from fruit, frozen in liquid nitrogen and stored at −80 °C until analysed. Three biological replicates were performed for analyses at each sampling point.

Naringenin, malonyl‐CoA and other flavonoid standards for measurement were purchased from Sigma‐Aldrich (https://www.sigmaaldrich.com). Naringenin chalcone, isoliquiritigenin, liquiritigenin and *p*‐coumaroyl‐CoA were purchased from Yuanye (http://www.shyuanye.com). All the chemicals were of HPLC (representative high‐performance liquid chromatography) grade.

### RNA extraction and RNA‐seq

Total RNA was extracted from frozen citrus samples according to a previous protocol (Chang *et al*., 1993). RNA isolated from the peels of four stages (S1, S3, S5 and S7) was used for sequencing. Library construction, RNA sequencing and bioinformatics analyses were performed by Majorbio (Shanghai, China). The libraries were sequenced using the Illumina HiSeq2000 sequence platform, and the clean reads were mapped to the *Citrus Clementina* genome database (https://www.citrusgenomedb.org/). Gene transcript levels detected in RNA‐seq were estimated by FPKM values.

For *A. thaliana* plants, total RNA was isolated using the RNAiso Plus (Takara, Beijing, China) kit following the manufacturer’s protocol.

### cDNA synthesis and RT‐PCR

For both citrus and Arabidopsis, PrimeScript™ RT reagent Kit with gDNA Eraser (Takara, Beijing, China) was used to remove the genomic DNA contamination from total RNA and to synthesize the cDNA. The RT‐PCR analyses were conducted following the previous protocol described by Liu *et al*. (2020). Three independent biological replicates were assayed for each quantitative PCR experiment. Primers used for RT‐PCR are listed in Table S3, and the specificity of primers was examined by melting curves and products resequencing.

### Flavonoid extraction and measurement

The citrus flavonoids were extracted as described in a previous study (Liu *et al*., 2020) with minor modification. An HPLC‐DAD system (2695 quaternary pump, 2996 diode array detector; Waters Crop., Milford MA) coupled with a Sunfire C18 ODS column (4.6 × 250 mm, 5 μm; Waters.) was used for individual flavonoid detection. The compounds were separated at room temperature using the following gradients of acetonitrile (solvent A) versus 0.1% (v/v) formic acid in water (solvent B) at a flow rate of 1 mL/min: 0–5 min, 20% A; 5–10 min, 20%–27% A; 10–15 min, 27% A; 15–25 min, 27%–40% A; 25–35 min, 40%–60% A; 35–40 min, 60%–80% A; 40–42 min, 80%–100% A; 42–45 min, 100%–20% A; 45–50 min, 20% A (Liu *et al*., 2020). Flavonoids were monitored at 200–400 nm, and the concentration of individual flavonoids was calculated by using a standard curve method.

The Arabidopsis metabolites were extracted and hydrolysed following the protocol described by Jiang (Jiang *et al*., 2015). 0.1 g of Arabidopsis samples was extracted by 1 mL 80% methanol twice, and 200 μL 6 N HCl was added into 200 μL extraction and incubated at 70 °C for 1 h for hydrolysation. After hydrolysis, 400 μL methanol was added to dilute the HCl, and the flavonols were detected by HPLC.

### Genes isolation, promoter cloning and plasmid construction

The full‐length coding sequences (CDS) of all the genes used in this study were amplified from the Ougan cDNA library and cloned into the pMD19‐T vectors (Takara, Beijing, China) for resequencing. The promoter fragment of *CitCHIL1* (~2.1 kb) was amplified from Ougan genomic DNA and the sequence verified as described above. The genomic DNA of Ougan was extracted from leaves using EZ‐10 Spin Column Plant Genomic DNA Purification Kit (Sangon Biotech, Shanghai, China).

For expression of recombinant proteins, the full‐length open reading frames (ORFs) of *CitCHIL1*, *CitCHI*, *CitCHS1*, *CitCHS2*, *CitERF32*, *CitERF33* and *CitRAV1* were inserted into pET32a vector to generate N‐ and C‐terminal histidine‐fused proteins. The plasmids were transformed into *E. coli* strain BL21 (DE3) pLysS (Promega).

For subcellular localization, the ORF of the genes (*CitCHI*, *CitCHIL1*, *CitCHS1*, *CitCHS2*, *CitERF32*, *CitERF33* and *CitRAV1*) was inserted into the pCAMBIA1300‐eGFP vector, and all the constructs were transferred into *Agrobacterium tumefaciens* strain GV3101 by electroporation.

For Y2H assays, the ORF of CitCHIL1 was inserted into pGBKT7 vector and the ORFs of *CitCHS1*, *CitCHS2* and *CitCHI* were inserted into pGADT7 vector.

For BiFC assays, the ORFs of target genes (*CitCHI*, *CitCHIL1*, *CitCHS1*, *CitCHS2*, *CitERF32*, *CitERF33* and *CitRAV1*) were cloned into the YFP^N^ (p2YN) and YFP^C^ (p2YC) vector and the constructs were transferred individually into *A. tumefaciens* strain GV3101.

For LCI assays, the ORFs of *CitCHI*, *CitCHS1*, *CitCHS2*, *CitCHIL1* and *AtCHS* were cloned into pCAMBIA1300‐cLUC (cLUC) and pCAMBIA1300‐nLUC (nLUC) vectors.

For overexpression *in vivo*, the full‐length ORFs of target genes (CitCHIL1, CitERF32, CitERF33, CitRAV1) were inserted into pBI121 vector and the constructs were transferred into *A. tumefaciens* strain EHA105 (for expression in citrus) or GV3101 (for transformation in Arabidopsis).

For dual‐luciferase assays, the promoter of *CitCHIL1* was inserted into the pGreen II LUC vector (LUC) and the TFs were inserted into the pGreen II SK vector (SK), respectively.

For VIGS, a 300‐bp length sequence amplified from CDS of CitCHIL1 was inserted into the TRV2 vector. The TRV1 and constructed TRV2 vectors were transferred into *A. tumefaciens* strain EHA105.

For GST pull‐down assays, the ORF of *CitERF33* was inserted into a pGEX‐4t‐1 vector, and the plasmid was transferred into *E. coli* strain BL21 (DE3) pLysS.

All the primers used for plasmid constructions are listed in Table S3.

### Expression and purification of recombinant proteins

The recombinant proteins were expressed and purified as described by Li *et al*. (2017). All the proteins were analysed by SDS‐PAGE and the buffer exchanged through a PD‐10 Desalting Column (GE Healthcare, Uppsala, Sweden) for subsequent storage.

The purified proteins were stored at −80 °C and the following storage buffers: 100 mm HEPES‐KOH buffer (10% glycerol, 2 mm DTT; pH 7.0) for CHS proteins; 50 mm potassium phosphate buffer (10% glycerol, 2 mm DTT; pH 7.5) for CHI proteins; and 50 mm Tris‐HCl buffer (10% glycerol, 2 mm DTT; pH 8.0) for transcription factors.

### Enzyme assays and kinetic determination

The purified proteins were quantified using a Modified BCA Protein Assay Kit (Sangon Biotech, Shanghai, China). The chalcone isomerase assays were performed in a 500 μL volume containing 50 mm potassium phosphate buffer (pH 7.5), 2 mm DTT and 50 μg purified CHIL1 protein, 200 μm substrate and 5% (v/v) ethanol. The reactions and product extraction were conducted as described by Cheng *et al*. (2018). The products were analysed by an ultra‐performance liquid chromatography‐diode array detector (UPLC‐DAD, Waters) coupled with ACQUITY UPLC^®^ HSS T3 column (2.1 × 100 mm, 1.8 μm; Waters). The column was operated at room temperature at a flow rate of 0.2 mL/min. The gradient programme of the mobile phase was set as: 0–2 min, 40% A (methanol)/60% B (water); 2–10 min, 40%–80% A; 10–15 min, 80%–100% A; 15‐23 min, 100%–40% A; and 23–27 min, 40% A/60% B. Mass spectrometric analyses were conducted on an Agilent 6460 triple‐quadrupole mass spectrometer equipped with an electron spray ion (ESI) source.

For determination of the stereochemistry of CHIL1 reaction products, a 100 mL volume reaction containing 5 mg naringenin chalcone and an excess amount of protein was incubated at room temperature. The products were extracted and re‐dissolved in methanol with final concentration c. 1.0 mg/mL. The samples (10 μL) were loaded on the HPLC‐DAD system for analyses using a CHIRALPAK^®^ IG column (4.6 × 250 mm, 5 μm; Daicel). The mobile phase was 100% methanol at a flow rate of 1.0 mL/min at room temperature.

A standard chalcone synthase assay was performed in a 500 μL reaction volume containing 100 mm HEPES‐KOH buffer (pH 7.0), 2 mm DTT, 5 μg purified CHS protein, with or without 4 μg purified CHIL1 protein. The reactions were conducted according to the procedure described by Ban (Ban *et al*., 2018). The products were extracted and analysed by UPLC as described above.

### Subcellular localization

*Agrobacterium tumefaciens* strains carrying the GFP constructs were grown on LB medium for over 48 h and then resuspended in infiltration buffer (10 mm MgCl_2_, 10 mm MES, 150 mm acetosyringone; pH 5.6) at an OD_600_ of 0.75. The suspension was infiltrated into the abaxial side of *N. benthamiana* leaves. Fluorescence was observed 3 days after transfection using a confocal laser scanning microscope (Zeiss LSM 710 NLO, Oberkochen, Germany).

### Yeast two‐hybrid assays

Y2H assays were conducted based on a Matchmaker Gold Yeast Two‐Hybrid System (Takara, Beijing, China). The positive transformants were selected on SD/‐Trp/‐Leu medium and suspended in 0.9% NaCl at OD_600_ = 0.5. To validate the protein–protein interactions, 20 μL of the suspension was spread on SD/‐His/‐Leu/‐Trp medium with 3‐AT and the SD/‐His/‐Leu/‐Trp/‐Ade medium with AbA. The plates were photographed after incubation at 30 °C for 5 days.

### Bimolecular fluorescence complementation assay

*Agrobacterium tumefaciens* harbouring the constructs of the YFP^N^ and YFP^C^ were mixed at a ratio of 1: 1 in infiltration buffer and injected into *N. benthamiana* leaves to generate the N‐terminal and C‐terminal YFP fusion protein. The infiltration in *N. benthamiana* leaves and imaging were carried out as described for subcellular localization studies.

### Firefly luciferase complementation imaging assay

*Agrobacterium tumefaciens* strain GV3101 carrying the constructs of nLUC and cLUC were mixed and infiltrated into *N. benthamiana* leaves. The LUC activity was determined 2 days after infiltration. Before imaging, 0.2 mm luciferin was injected to the position infiltrated by *A. tumefaciens* and held for 15 min in the dark. The LUC imaging was performed using the NightSHADE LB 985 system.

### Transient overexpression in citrus fruit and leaves

*Agrobacterium tumefaciens* cultures were prepared using the same method detailed for subcellular localization. For citrus leaves, the suspensions expressing target genes and empty pBI121 vector (as control) were injected in different sides of the same leaf on opposite sides of the main vein (Li *et al*., 2017). For citrus fruit, the suspensions carrying control and target genes were injected into the peel on opposite sides of the equatorial plane of the same fruit (Yin *et al*., 2016). The fruit of Ougan used for infiltration was harvested from an orchard in Hangzhou, China, at 170 DAFB, and the Ougan seedlings used for transient transformation in leaves were maintained under a 16‐h light/8‐h dark cycle at 25 °C. After infiltration, the seedlings and fruits were kept in the dark for 1 day and maintained under 16‐h light/8‐h dark condition. The metabolites were analysed at 5 days after infiltration and measured by HPLC.

### Transformation and analyses in *Arabidopsis*

Four‐week‐old seedlings of WT *A. thaliana* ecotype Columbia‐0 were infiltrated with the *A. tumefaciens* (GV3101) suspension carrying the *CitCHIL1*‐pBI121 construct using the floral‐dip method (Clough and Bent, 1998). After selection on 1/2 MS medium with 50 mg/mL kanamycin and 25 mg/mL meropenem, the transformants were moved to moistened soil and grown in a greenhouse at 23 °C under a 16‐h light/8‐h dark cycle.

The siliques of 5‐week‐old seedlings (T_3_ and WT) were collected and analysed.

### TRV‑mediated VIGS in *Citrus*

The TRV‐mediated VIGS was performed according to Wang (Wang *et al*., 2017a). *Agrobacterium* cells carrying TRV1 and TRV2 constructs were grown in LB broth to an OD_600_ = 1.0, and then, the cultures of *Agrobacterium* were centrifuged at 9300 *g* for 10 min and resuspended in the infiltration buffer (10 mm MgCl_2_, 10 mm MES, 200 mm acetosyringone; pH 5.6). The resuspended *Agrobacterium* with TRV1 and TRV2 constructs were mixed in 1:1 volume ratio and infiltrated into germinated seedlings of Jincheng by vacuum method. After infiltration, the seedling was grown in darkness for 2 days and then moved to a growth chamber for 1 month. The flavonoid contents and gene expression were determined as described above.

### GUS staining

Histochemical staining of transformed *Arabidopsis* was performed using a GUS Staining Kit (Real‐Times, Beijing, China). Three‐week‐old seedlings of *Arabidopsis* were immersed into the X‐gluc buffer and infiltrated with staining buffer under vacuum. After incubation at 37 °C overnight, the seedlings were destained by ethanol and photographed with a binocular stereomicroscope (Carl Zeiss, Oberkochen, Germany).

### Dual‐luciferase assays

Dual‐luciferase assays were conducted following the procedure used by Li *et al*. (2017). *A. tumefaciens* (strain GV3101) cultures harbouring TF‐SK plasmids and promoter‐LUC plasmids were mixed at a ratio of 10: 1(v/v) in infiltration buffer, and the mixtures were injected into *N. benthamiana* leaves. The luminescence from Firefly luciferase (LUC) and Renilla luciferase (REN) was detected by Dual‐Luciferase Reporter Assay System (Promega, Madison, WI) on the third day after infiltration.

### Electrophoretic mobility shift assay

The recombinant proteins were obtained as described above, and the probes with or without 3' biotin end‐labelled were synthesized by Invitrogen (Thermo Fisher Scientific). The electrophoretic mobility shift assays (EMSA) were performed using the LightShift Chemiluminescent EMSA Kit (Thermo Fisher Scientific) following the user manual.

### Pull‐down assay

*Escherichia coli* harbouring CitERF33‐GST was induced by 0.1 mm IPTG and incubated at 20 °C for 3 h. The crude GST‐fused protein was extracted by ultrasonication and centrifugation. The supernatant containing the recombinant proteins was incubated with the GST resins for 2 h for GST protein immobilization, and the purified CitRAV1‐His protein was added subsequently and incubated with the immobilized protein overnight at 4 °C. After incubation, the resins were washed three times and the immobilized proteins were eluted by GSH. The eluted proteins were analysed by Western blot with anti‐His, anti‐GST (TransGen, Beijing, China).

### Statistics

All experiments in this study were repeated at least three times. Figures were plotted by Origin2019 (Microcal Software Inc., Northampton, MA) with error bars representing standard error (SE). The data were statistically assessed using SPSS version 20.0. Statistical significance of differences was calculated using one‐way ANOVA and tested with a confidence level of 95.0% (*, *P* < 0.05), 99.0% (**, *P* < 0.01) or 99.9% (***, *P* < 0.001).

## Conflict of Interest

The authors declare that they have no conflict of interest.

## Author contributions

C.S. conceived the research plans; C.Z. performed most of the experiments and analyses with helps from X‐J.L. and Q.G.; W.S. provided technical assistance to C.Z. on the RNAi manipulations; C.Z., J.C. and C.S. wrote the original draft; C.S., X.Y., D.G., C.X., B.Z., X.L. and K.C. reviewed and edited the manuscript. All authors have read and agreed to the published version of the manuscript.

## Supporting information


**Figure S1.** Main flavonoids in citrus and their biosynthetic pathways.
**Figure S2.** The correlation between expression of CHI‐fold genes and flavonoid content during fruit development.
**Figure S3.** Sequence alignment of CitCHIL1 with type IV CHI from other species and a *bona fide* chalcone isomerase AtCHI.
**Figure S4.** Analysis of recombinant proteins and chalcone isomerase reactions.
**Figure S5.** Kinetic study of CitCHSs and CitCHS/CitCHIL1 complexes.
**Figure S6.** Physical interaction between CitCHIL1 and AtCHS and main flavonoids in Arabidopsis.
**Figure S7.** Regulatory effects of TFs screened by Y1H library screening with the promoter of *CitCHIL1*.
**Figure S8.** Dual‐luciferase assays and schematic representations of *CitCHIL1* promoter deletion.
**Figure S9.** The original Western blot image of pull‐down assay.
**Table S1.** TFs screened by Y1H library screening.
**Table S2.** Potential TFs predicted to interact with CitCHIL promoter (Prediction by PlantTFDB).
**Table S3.** Primers used in the present study.
