# Supplementary material for: Three AP2/ERF family members modulate flavonoid synthesis by regulating type IV chalcone isomerase in citrus
Source: Plant Biotechnol J. 2020 Nov 27;19(4):671–88. doi: 10.1111/pbi.13494 (PMC8051604; doi:10.1111/pbi.13494)
Supplement: Supplementary file 1 — Figure S1. Main flavonoids in citrus and their biosynthetic pathways. Figure S2. The correlation between expression of CHI‐fold genes and flavonoid content during fruit development. Figure S3. Sequence alignment of CitCHIL1 with type IV CHI from other species and a bona fide chalcone isomerase AtCHI. Figure S4. Analysis of recombinant proteins and chalcone isomerase reactions. Figure S5. Kinetic study of CitCHSs and CitCHS/CitCHIL1 complexes. Figure S6. Physical interaction between CitCHIL1 and AtCHS and main flavonoids in Arabidopsis. Figure S7. Regulatory effects of TFs screened by Y1H library screening with the promoter of CitCHIL1. Figure S8. Dual‐luciferase assays and schematic representations of CitCHIL1 promoter deletion. Figure S9. The original Western blot image of pull‐down assay. Table S1. TFs screened by Y1H library screening. Table S2. Potential TFs predicted to interact with CitCHIL promoter (Prediction by PlantTFDB). Table S3. Primers used in the present study. [file PBI-19-671-s001.pdf]

Figure S1. Main flavonoids in citrus and their biosynthetic pathways.

(a) The proposed early biosynthetic pathways of flavanones and flavones in citrus. (**b)** Representative high-performance liquid chromatography (HPLC) profiles of flavonoid detected in the peel of stage 1 (S1) fruit at 280nm. (Eriocitrin and Neoeriocitrin mainly accumulate in leaves).

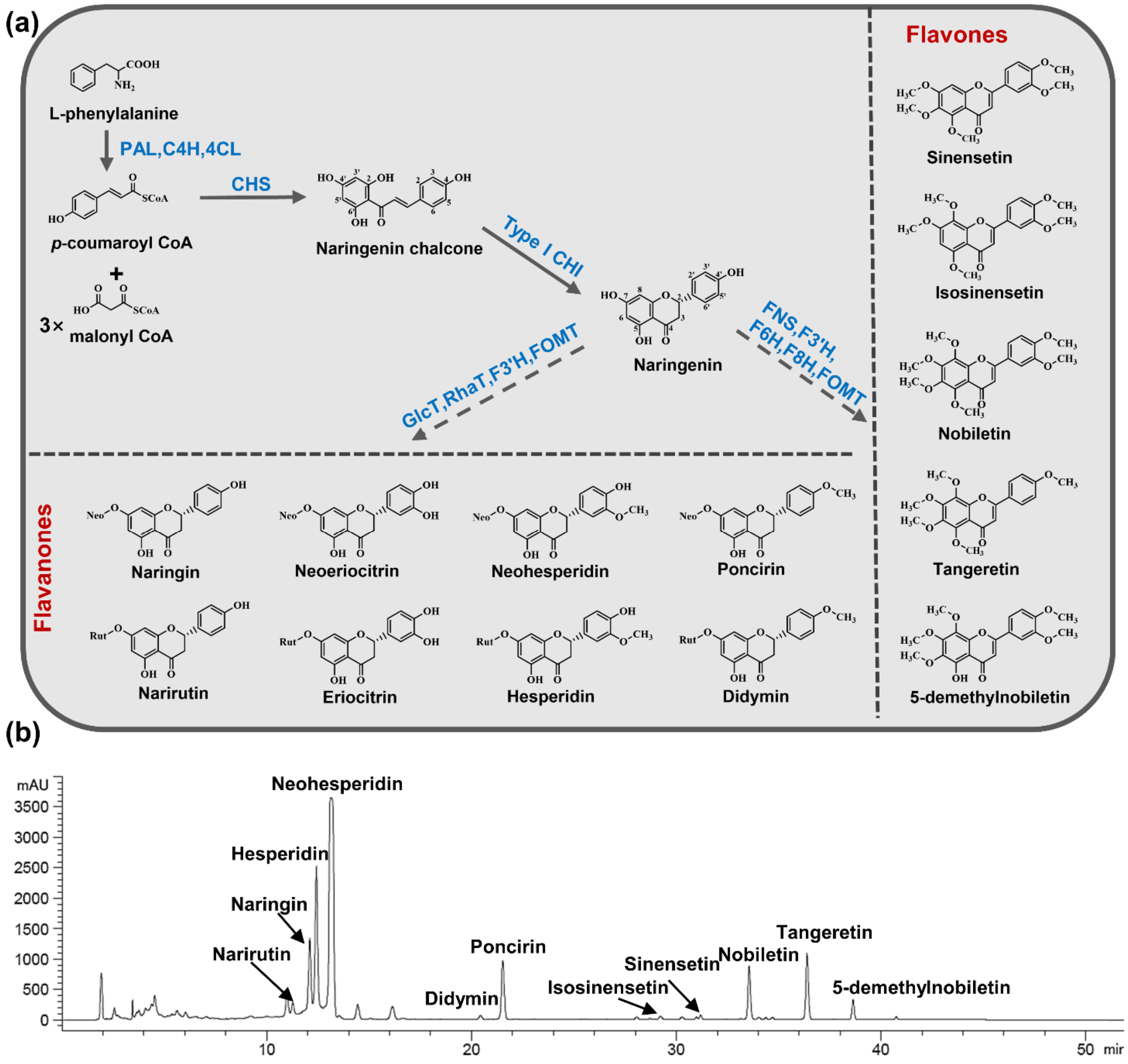

Figure S2. The correlation between expression of *CHI-fold* genes and flavonoid content during fruit development. The linear fitting analysis were conducted by Origin Pro with significant differences obtained by *F*-test (ANOVA).

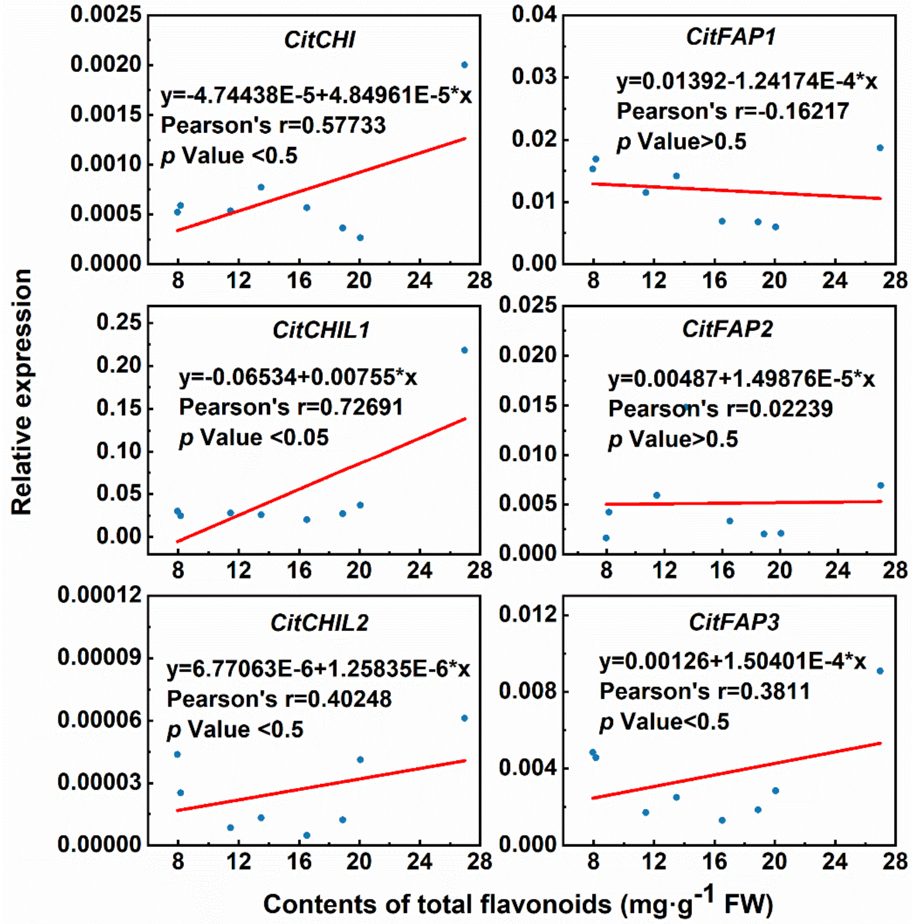

Figure S3. Sequence alignment of CitCHIL1 with type IV CHI from other species and a *bona fide* chalcone isomerase AtCHI.

The structure of AtCHI (PDB: 4DOI) in RCSB PDB (http://www.rcsb.org/) was used as protein template (Ngaki et al., 2012). Secondary structure is displayed above the sequences block. Helices and β-strands are depicted as coils and arrows. Residues of the (*2S*)-naringenin binding cleft are shown with yellow stars, and those for the hydrogen bond network in the active site are shown with red stars (Cheng et al., 2018). The yellow box identifies residues proposed to determine substrate preference for isoliquiritigenin and naringenin chalcone. The conserved sequence alignment of type IV chalcone isomerase branch was analyzed using Expresso method in T-Coffee (http://tcoffee.crg.cat/) and the protein structures were marked by ESPript 3.0 (http://espript.ibcp.fr/).

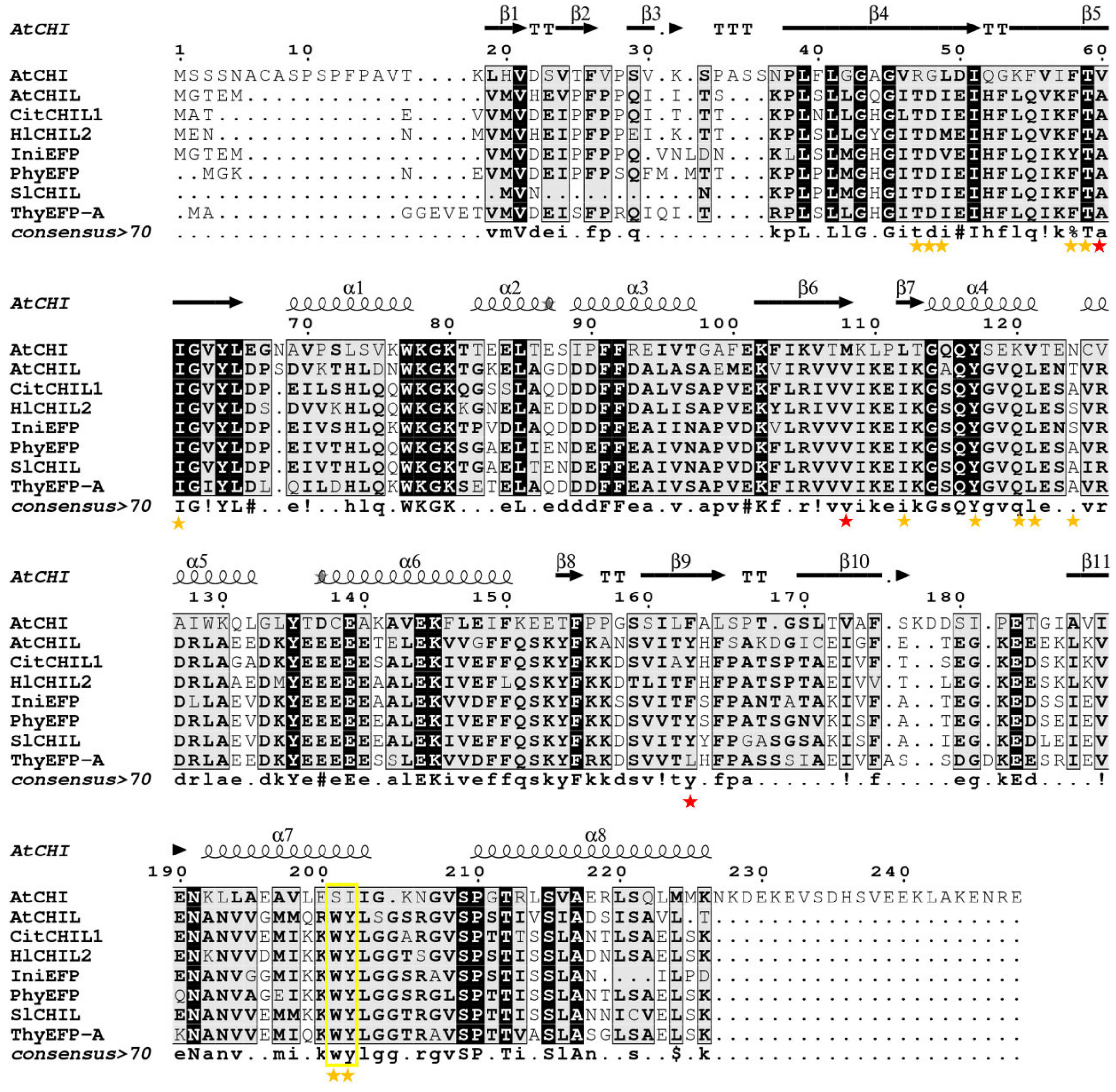

Figure S4. Analysis of recombinant proteins and chalcone isomerase reactions.

(a) SDS-PAGE analysis of the recombinant enzymes expressed in *E. coli* strain BL21. C. E., crude extract; P. P., purified protein; M, protein marker. **(b, c)** HPLC analyses of the products from chalcone isomerase reaction. **(b)** Reactions incubated with naringenin chalcone as substrate and using 2μg protein. NC, naringenin chalcone; NA, naringenin; EV, proteins of empty pET32a vector. **(c)** Reactions incubated with isoliquiritigenin as substrate and using 50μg protein. ILQ, isoliquiritigenin; LQ, liquiritigenin; EV, proteins of empty pET32a vector.

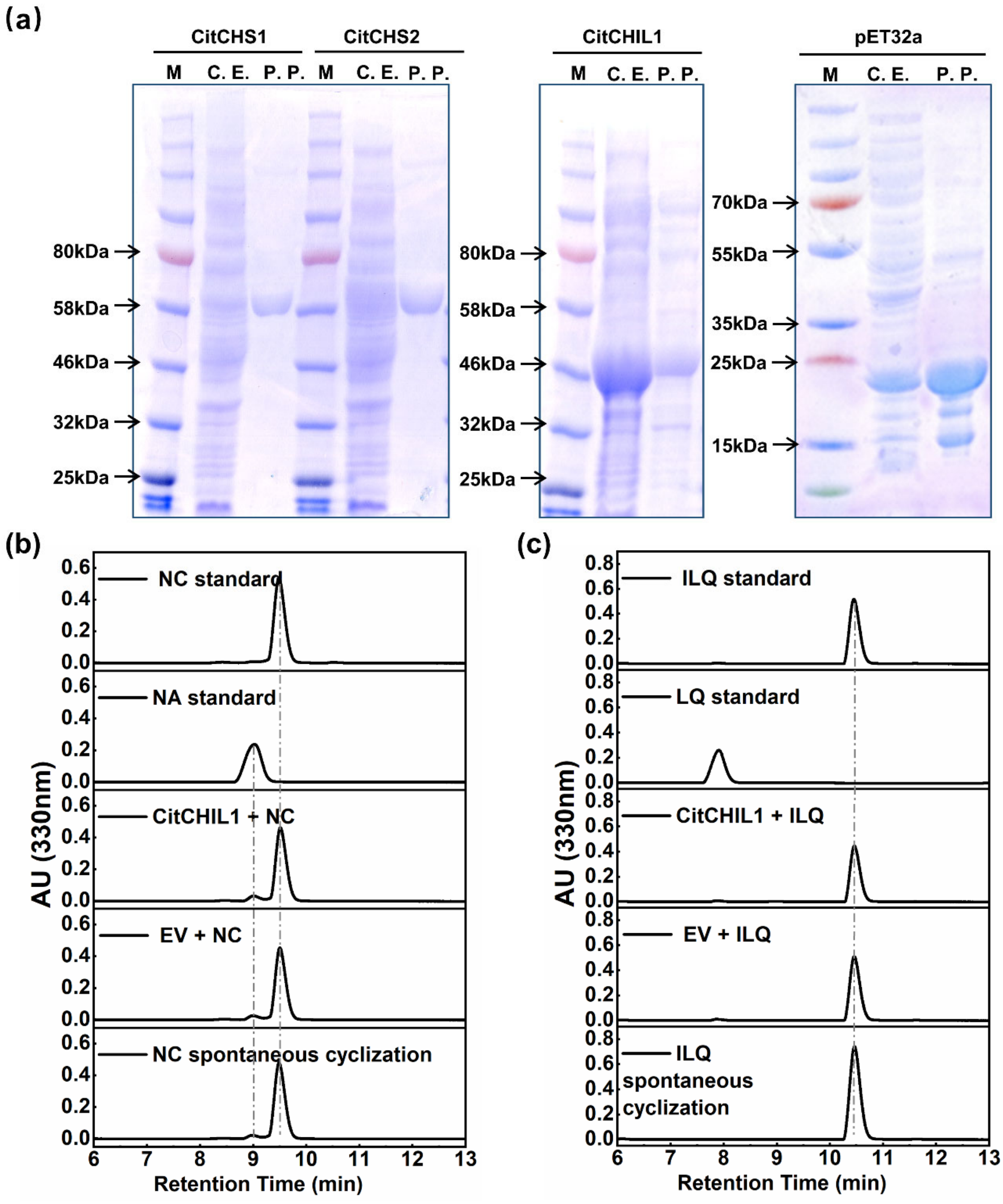

Figure S5. Kinetic study of CitCHSs and CitCHS/CitCHIL1 complexes.

Michaelis-Menten plots of CitCHS1 and CitCHS1/CitCHIL1 complex and CitCHS2 and CitCHS2/CitCHIL1 complex with inset Lineweaver-Burk plots. The kinetic parameters K_m and V_{max} were calculated by nonlinear regression analysis using GraphPad Prism 8 software. These results were averages of three experiments. Error bars denote SE of the means.

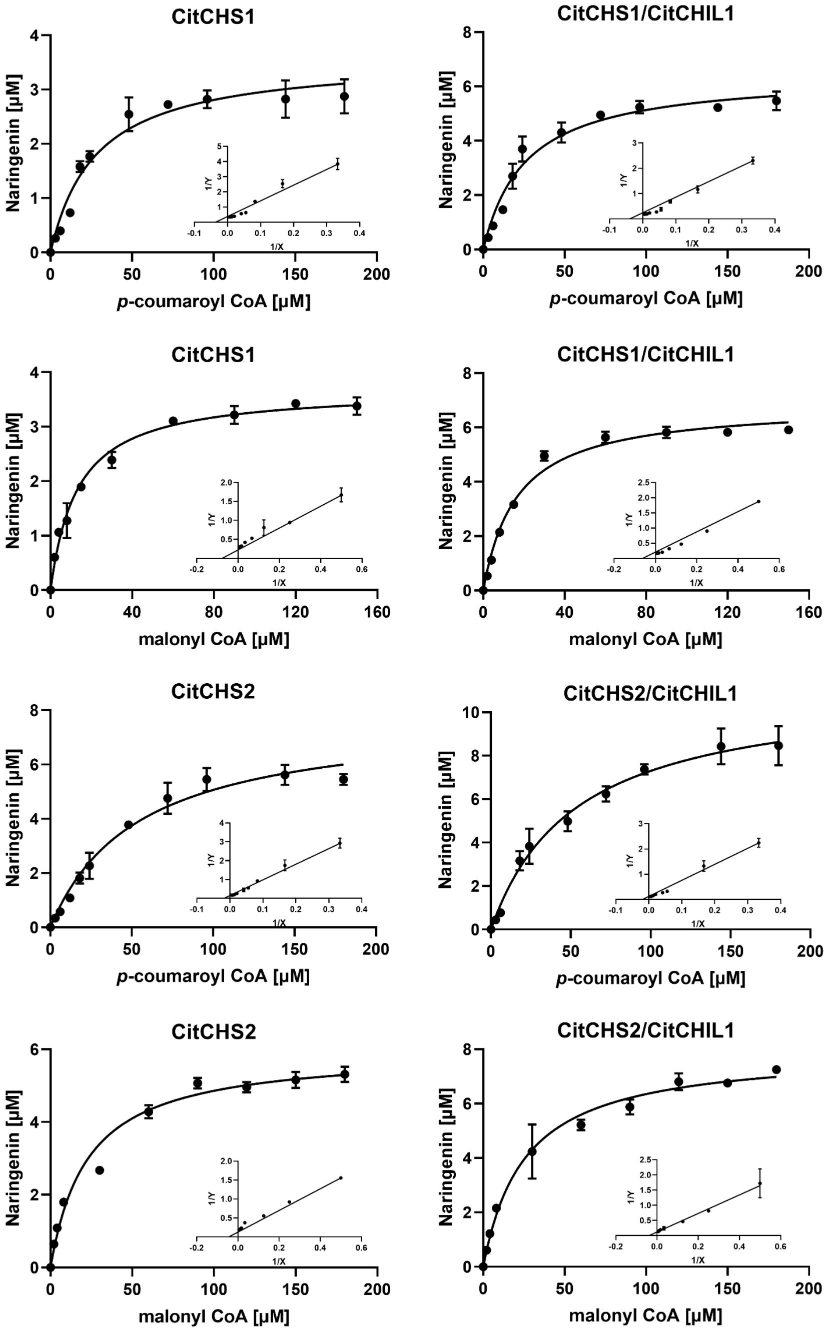

Figure S6. **Physical interaction between CitCHIL1 and AtCHS and main flavonoids in Arabidopsis.**

(a) Firefly luciferase complementation imaging (LCI) analyses of the direct interaction between CitCHIL1 and AtCHS in *Nicotiana benthamiana* leaves. The luciferase images visualized the interaction *in vivo*. (b) Representative HPLC profiles of flavonoid detected in the silique of 5-week-old WT and transgenic Arabidopsis at 350nm.

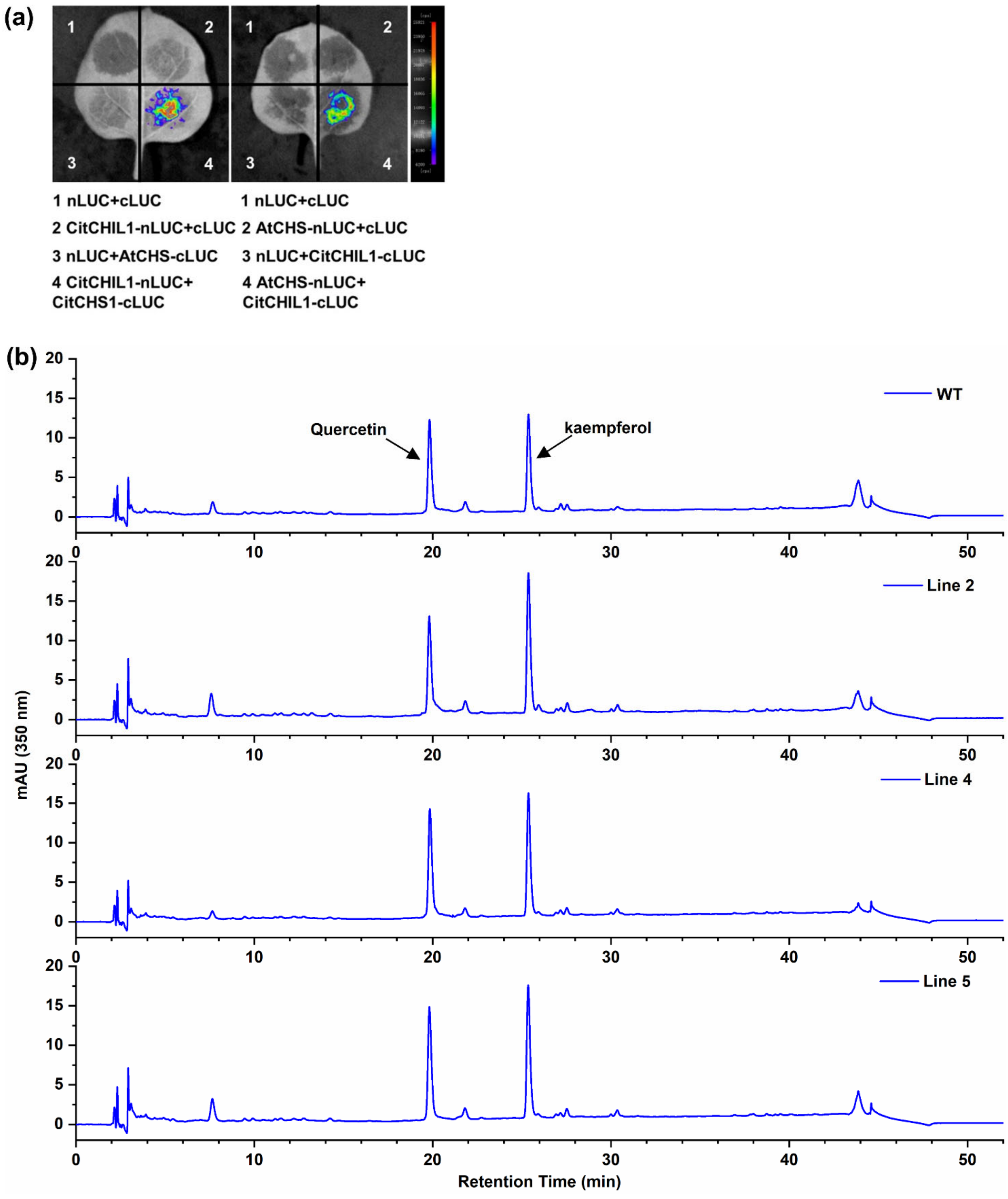

Figure S7. Regulatory effects of TFs screened by Y1H library screening with the promoter of *CitCHIL1*. Firefly luciferase/Renilla luciferase (LUC/REN) values of the empty pGreenII-SK vector on the *CitCHIL1* promoter were set as 1, and SE values were calculated from six replicates. Statistical significance was determined by one-way ANOVA and LSD test.

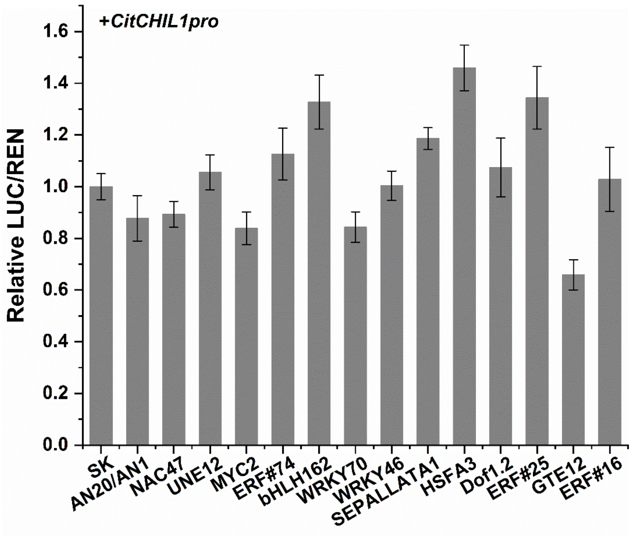

Figure S8. Dual-luciferase assays and schematic representations of *CitCHIL1* promoter deletion. The promoter was truncated to 1000bp length (pro1.0), 500bp (pro0.5) and 200bp (pro0.2), respectively. Error bars denote SE of the means (n = 6). Each TFs-promoter interaction was confirmed by three independent experiments and each experimental point had at least six biological replicates.

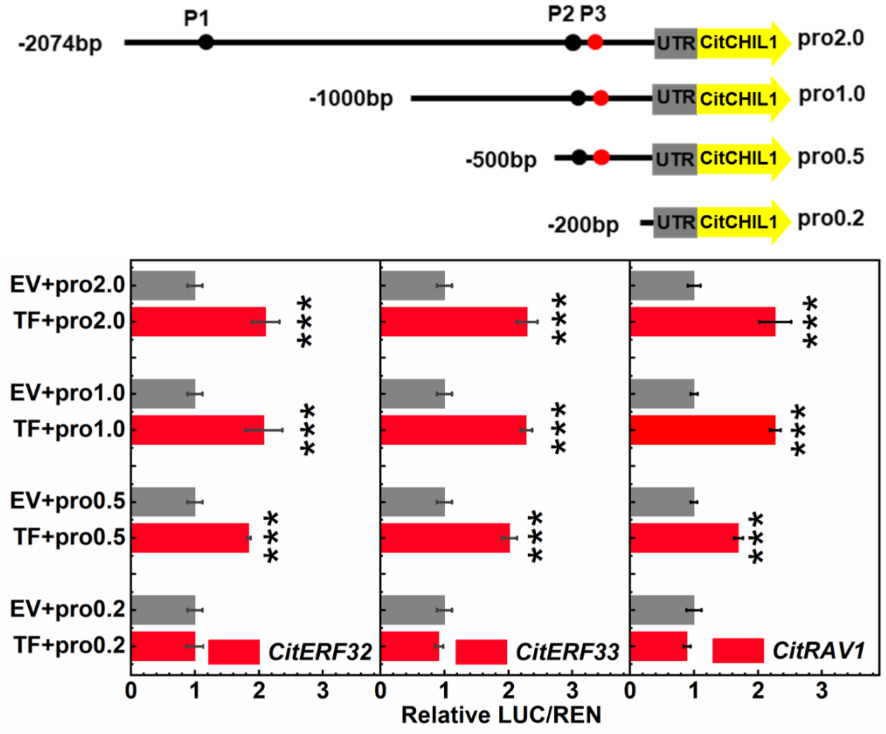

Figure S9. The original Western blot image of pull-down assay.

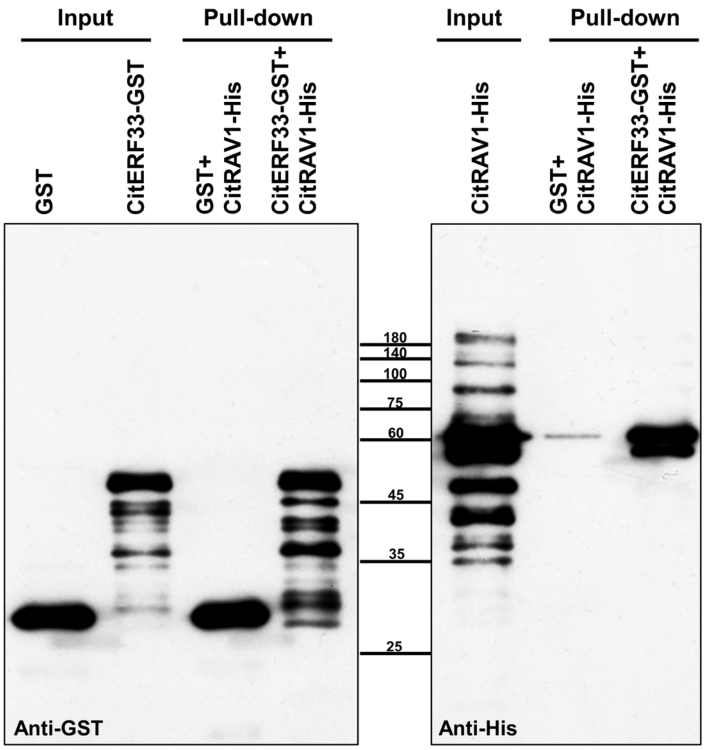

Table S1. TFs screened by Y1H library screening.

System: Matchmaker™ One-Hybrid Library Screening Kit (Clontech,Takara)

Library: Ougan Fruit and Leaves

Bait (Promoter Regions): Region A (-2074bp~-1355bp); Region B (-1436bp~-725bp); Region C (-806bp~-1bp)

Remarks: All the regions have self-activating effect (AbA: 250ng/ml)

No.	Region of Promoter	RecName	Short Name	GenBank	Gene ID	Frequ-ency
1	C	A20/AN1-like zinc finger family protein	AN20/AN1	XM_006430173.2	Ciclev10012937m.g	1
2	C	NAC domain containing protein 47	NAC47	XM_006478987.3	Ciclev10020717m.g	1
3	C	basic helix-loop-helix (bHLH) DNA-binding superfamily protein (UNE12)	UNE12	XM_025096319.1	Ciclev10001768m.g	1
4	C	Basic helix-loop-helix (bHLH) DNA-binding family protein MYC2	MYC2	XM_006491671.3	Ciclev10011214m.g	1
5	B	ethylene-responsive element binding factor 13	ERF#74	XM_006445353.2	Ciclev10022734m.g	1
6	B	basic helix-loop-helix (bHLH) DNA-binding superfamily protein 162	bHLH162	XM_006447229.2	Ciclev10016466m.g	2
7	B	WRKY DNA-binding protein 70	WRKY70	XM_006435880.2	Ciclev10032192m.g	1
8	B	WRKY DNA-binding protein 46	WRKY46	XM_006444813.2	Ciclev10020744m.g	1
9	B	K-box region and MADS-box transcription factor family protein	SEPALLATA1	XM_006430886.2	Ciclev10012593m.g	1
10	B	heat shock transcription factor A3	HSFA3	XR_002904938.1	Ciclev10011698m.g	1
11	C	Citrus clementina dof zinc finger protein DOF1.2	DOF1.2	XM_006434360.2	Ciclev10002179m.g	1
12	C	Citrus clementina ethylene-responsive transcription factor ERF023	ERF#25	XM_006441744.2	Ciclev10022438m.g	1
13	C	Citrus clementina transcription factor GTE12	GTE12	XM_006449532.2	Ciclev10014673.g	1
14	C	C-repeat/DRE binding factor 2	ERF#16	XM_006440466.2	Ciclev10021923.g	1

Table S2. Potential TFs predicted to interact with CitCHIL promoter. (Prediction by PlantTFDB)

[a] The data are presented as means±SE (n = 3).

[b] Correlation coefficient (with *CitCHIL*); The Linear regression analyses between CitCHIL expression and TFs expression were conducted by OriginPro with significant differences obtained by *F* -test (ANOVA). *, $p<0.05$; **, $p<0.01$

[c] The AP2/ERF family genes are named according to the previous report (Xie et al., 2014).

[d]MYB and MYB-related genes are with pale yellow background.

No.	tracking_id	subfamily	FPKM value				r[b]	Short Name
			S1	S3	S5	S7		
1	Ciclev10012205m.g	bZIP	5.45±0.69[a]	0.65±0.15	0.34±0.14	0.42±0.03	0.9941**	*CitABF2*
2	Ciclev10021911m.g	MIKC_MADS	41.45±2.45	9.99±2	8.42±0.53	8.38±0.7	0.9932**	*CitAGL6*
3	Ciclev10005549m.g	ERF	1.92±0.3	0.06±0.03	0.15±0.08	0.09±0.05	0.9931**	*CitERF32* [c]
4	Ciclev10002280m.g	ERF	4.83±0.86	0.25±0.07	0.21±0.11	0.11±0.03	0.9931**	*CitERF33* [c]
5	Ciclev10025111m.g	AP2	13.92±1.34	1.41±0.23	0.5±0.12	0.49±0.11	0.9917**	*CitAP2-5* [c]
6	Ciclev10001165m.g	E2F/DP	2.27±0.34	0.93±0.25	0.99±0.32	1.24±0.2	0.9907	(did not get full-length gene)
7	Ciclev10015354m.g	BBR-BPC	133.84±3	46.74±0.37	33.85±3.29	38.44±0.87	0.9902	(did not get full-length gene)
8	Ciclev10032076m.g	bZIP	51.1±2.1	14.44±0.62	18.77±1.85	26.07±2.17	0.9743*	*CitRF2b*
9	Ciclev10012003m.g	MYB[d]	9.4±0.2	5.49±0.13	4.04±0.48	4.88±0.24	0.9716*	*CitMYB44*
10	Ciclev10016427m.g	MIKC_MADS	188.84±5.96	107.01±10.88	81.79±3.42	74.67±2.58	0.9484	*CitMADS1*
11	Ciclev10015986m.g	MYB	7.89±5.56	3.8±0.3	4.95±0.52	5.37±1.2	0.9373	*CitMYB108*
12	Ciclev10020499m.g	C2H2	9.44±0.75	4.71±0.41	2.2±0.38	1.53±0.06	0.9034	*CitAZF2*
13	Ciclev10031846m.g	RAV	819.02±39.76	346.98±80.08	389.88±35.26	143.68±20.21	0.8857	*CitRAV1* [c]
14	Ciclev10015695m.g	Trihelix	9.6±0.61	5.78±0.34	3.33±0.28	2.74±0.12	0.8854	*CitGT-2*
15	Ciclev10032279m.g	HD-ZIP	32.82±1.92	10.05±0.79	9.42±1.45	23.02±0.63	0.8782	*CitATHB-13*
16	Ciclev10005233m.g	bHLH	33.24±3.41	19.31±3.65	5.29±0.7	16.32±1.86	0.8751	*CitbHLH130*
17	Ciclev10005411m.g	MYB_related	58.44±3.38	32.66±3.48	37.41±5.9	20.39±1.51	0.8450	*CitREVEILLE6*
18	Ciclev10031028m.g	bHLH	1.41±0.21	0.79±0.24	0.18±0.04	0.08±0.04	0.8389	(did not get full-length gene)
19	Ciclev10019949m.g	Dof	14.12±1.26	6.49±1.22	2±0.32	9.71±1.51	0.8378	*CitDof5.2*
20	Ciclev10001249m.g	bHLH	15±0.89	4.92±0.63	9.35±0.73	11.42±0.97	0.7947	*CitbHLH74*
21	Ciclev10026245m.g	MYB_related	23.38±1.16	20.31±3.02	13.96±1.33	15.06±0.55	0.7718	*CitSMH3*
22	Ciclev10006161m.g	GATA	25.94±1.12	21.69±3.75	9.21±0.57	15.73±1.4	0.7299	*CitGATA16*
23	Ciclev10021073m.g	ERF	2.56±0.2	1.77±0.08	0.97±0.15	0.08±0.01	0.7112	*CitERF48* [c]
24	Ciclev10000593m.g	Trihelix	53.44±2.2	40.24±0.19	22.92±3.9	47.19±3.16	0.6937	
25	Ciclev10026061m.g	Dof	53.35±0.78	46.46±6.1	21.91±6.38	8.79±0.94	0.6186	
26	Ciclev10021100m.g	Dof	31.59±1.09	19.99±4.43	28.96±1.75	28.68±1.87	0.5817	

**27**	**Ciclev10026013m.g**	**HD-ZIP**	**302.75±12.37**	**292.92±23.02**	**201.49±20.2**	**168.24±5.92**	**0.5729**
**28**	**Ciclev10030860m.g**	**ARF**	**14.07±1.34**	**6.72±0.63**	**8.59±0.34**	**14.94±0.94**	**0.5643**
**29**	**Ciclev10032039m.g**	**Dof**	**3.06±0.13**	**3.06±0.24**	**1.3±0.45**	**1.07±0.13**	**0.5466**
**30**	**Ciclev10010579m.g**	**NAC**	**3.23±0.14**	**3.18±0.21**	**1.16±0.34**	**0.61±0.12**	**0.5447**
**31**	**Ciclev10007882m.g**	**WRKY**	**41.32±1.59**	**40.69±1.08**	**27.43±3.01**	**36.21±1.71**	**0.5439**
**32**	**Ciclev10031135m.g**	**bHLH**	**8.63±0.72**	**3.42±0.5**	**6.82±0.88**	**8.65±0.74**	**0.5199**
**33**	**Ciclev10025816m.g**	**ERF**	**616.7±2.93**	**404.93±39.1**	**613.99±77.98**	**369.72±6.13**	**0.5166**
**34**	**Ciclev10004533m.g**	**C2H2**	**24.12±0.83**	**24.95±1.36**	**11.97±1.66**	**15.68±1.35**	**0.5159**
**35**	**Ciclev10005387m.g**	**MYB**	**3.89±2.04**	**0.55±0.17**	**4.03±1.43**	**0.67±0.12**	**0.4965**
**36**	**Ciclev10000756m.g**	**MYB**	**9.56±0.25**	**8.98±0.71**	**8.06±1.36**	**5.29±0.09**	**0.4881**
**37**	**Ciclev10001051m.g**	**bZIP**	**15.94±1.19**	**16.88±1.49**	**7.86±1.2**	**7.61±0.83**	**0.4875**
**38**	**Ciclev10017466m.g**	**GRAS**	**36.23±0.96**	**41.7±0.09**	**9.63±3.28**	**14.73±1.44**	**0.4388**
**39**	**Ciclev10031622m.g**	**bHLH**	**265.9±16.58**	**68.75±11.23**	**301.89±98.01**	**24.95±1.49**	**0.4138**
**40**	**Ciclev10031405m.g**	**MYB**	**2.36±0.2**	**3.16±0.32**	**0.4±0.12**	**0.24±0.04**	**0.3494**
**41**	**Ciclev10021268m.g**	**MYB**	**4.15±0.27**	**4.56±0.41**	**1.65±0.59**	**4.02±0.37**	**0.3303**
**42**	**Ciclev10022568m.g**	**MIKC_MADS**	**0.49±0.06**	**0.61±0.26**	**0.27±0.01**	**0.05±0.03**	**0.2952**
**43**	**Ciclev10005102m.g**	**MYB**	**0.54±0.54**	**0.29±0.05**	**0.48±0.11**	**0.64±0.10**	**0.2822**
**44**	**Ciclev10008192m.g**	**bHLH**	**148.79±7.03**	**89.72±6.08**	**180.33±16.65**	**85.54±10.46**	**0.2596**
**45**	**Ciclev10026414m.g**	**HD-ZIP**	**72.67±34.66**	**100.39±15.6**	**43.07±7.09**	**15.2±5.28**	**0.2165**
**46**	**Ciclev10012016m.g**	**C2H2**	**37.17±0.67**	**43.26±3.54**	**27.5±1.62**	**33.11±2.03**	**0.1998**
**47**	**Ciclev10021278m.g**	**Dof**	**1.56±0.35**	**2.52±0.46**	**0.36±0.12**	**0.47±0.03**	**0.1945**
**48**	**Ciclev10032167m.g**	**MYB_related**	**6.64±0.51**	**7.54±0.8**	**3±1.22**	**9.24±1.79**	**0.0912**
**49**	**Ciclev10007904m.g**	**WRKY**	**37.32±0.23**	**36.78±1.82**	**28.25±6.72**	**47.6±0.26**	**0.0793**
**50**	**Ciclev10009593m.g**	**ERF**	**24.72±3.45**	**3.84±1.3**	**10.11±2.87**	**63.01±6.87**	**0.0717**
**51**	**Ciclev10027890m.g**	**bHLH**	**17.58±0.37**	**17.6±0.77**	**12.7±2.68**	**23.03±0.26**	**0.0687**
**52**	**Ciclev10012360m.g**	**C3H**	**25.8±1.32**	**24.15±1.61**	**32.16±4.66**	**15.63±0.71**	**0.0412**
**53**	**Ciclev10008390m.g**	**TCP**	**28.61±1.19**	**38.93±4.23**	**19.15±2.12**	**29.08±1.98**	**-0.0028**
**54**	**Ciclev10021151m.g**	**BES1**	**43.35±1.42**	**36.53±2.06**	**59.61±1.43**	**31.35±0.16**	**-0.0430**
**55**	**Ciclev10028398m.g**	**MYB**	**2.91±0.61**	**7.06±0.53**	**1.15±0.41**	**1.29±0.05**	**-0.0688**
**56**	**Ciclev10001679m.g**	**G2-like**	**4.57±0.59**	**3.37±0.3**	**8.75±0.95**	**1.51±0.09**	**-0.0775**
**57**	**Ciclev10020263m.g**	**bZIP**	**35.4±0.19**	**23.97±0.78**	**64.35±15.25**	**19.92±1.02**	**-0.0894**
**58**	**Ciclev10032412m.g**	**bHLH**	**13.21±0.65**	**5.82±0.06**	**35.92±8.04**	**3.94±0.24**	**-0.1388**

59	Ciclev10032192m.g	WRKY	85.8±6.98	250.32±34.51	50.97±8.37	28.92±6.08	-0.1532	
60	Ciclev10031606m.g	NAC	36.82±0.41	56.15±2.23	34.58±1.39	29.88±1.11	-0.1849	
61	Ciclev10028259m.g	AP2	58.75±2.19	54.52±1.4	150.03±31.26	21.31±0.96	-0.2300	
62	Ciclev10005376m.g	MYB	5.28±0.44	11.01±0.97	1.6±0.66	12.99±1.94	-0.2364	
63	Ciclev10008096m.g	G2-like	1.53±0.02	3.27±0.62	1.04±0.32	1.71±0.21	-0.2414	
64	Ciclev10005820m.g	ERF	3.63±2.42	21.95±6.48	1.86±0.83	1.36±0.17	-0.2657	
65	Ciclev10023345m.g	ERF	35.7±3.51	0.7±0.54	266.01±122.84	1.64±0.27	-0.2749	
66	Ciclev10005366m.g	TCP	7.2±0.41	5.96±0.89	9.93±0.76	30.58±1.74	-0.2755	
67	Ciclev10032816m.g	WRKY	202.13±6.38	182.37±4.69	240.72±54.46	248.06±6.64	-0.3113	
68	Ciclev10011214m.g	bHLH	112.98±0.98	226.96±26.74	179.56±4.01	54.09±2.48	-0.3459	
69	Ciclev10021174m.g	WRKY	51.58±1.85	41.43±0.63	363.58±107.38	35.45±1.15	-0.3604	
70	Ciclev10026105m.g	WRKY	37.35±4.19	23.82±10.44	599.35±228.13	3.62±0.67	-0.3613	
71	Ciclev10032029m.g	ERF	63.91±5.29	27.47±8.96	1238.03±304.78	17.96±2.32	-0.3657	
72	Ciclev10001400m.g	BBR-BPC	35.96±1.68	39.72±1.08	54.51±6.45	31.02±1.07	-0.3683	
73	Ciclev10020314m.g	bZIP	47.92±4.44	43.57±4.87	217.5±20.39	42.13±5.07	-0.3701	
74	Ciclev10014642m.g	WRKY	37.57±9.45	29.51±10.28	574.76±94.64	41.27±6.86	-0.3903	
75	Ciclev10026433m.g	MIKC_MADS	1.95±0.42	3.96±0.46	8.28±0.92	29.41±4.92	-0.3941	
76	Ciclev10019339m.g	bHLH	81.69±5.69	109.85±0.56	235.52±13.71	55.09±2.29	-0.4024	
77	Ciclev10008836m.g	WRKY	150.66±0.72	233.85±34.44	735.33±137.44	98.96±3.18	-0.4238	
78	Ciclev10032507m.g	MIKC_MADS	446.99±17.46	606.47±16.35	1030.18±25.47	2437.93±57.56	-0.4279	
79	Ciclev10020248m.g	bHLH	20.96±0.5	31.33±3.21	26.72±2.72	62.91±2.18	-0.4326	
80	Ciclev10033384m.g	WRKY	0.3±0.03	1.33±0.76	10.68±2.73	0.09±0.04	-0.4342	
81	Ciclev10028930m.g	ERF	7.65±3.07	104.04±36.88	975.04±93.81	22.07±3	-0.4495	
82	Ciclev10002294m.g	ERF	1.18±0.14	10.32±3.44	533.52±55.49	72.43±6.43	-0.4535	
83	Ciclev10001333m.g	bZIP	24.84±0.71	30.4±1.96	25.46±1.78	36.84±0.64	-0.4642	
84	Ciclev10028435m.g	bZIP	81±1.69	116.68±14.39	81.57±13.72	141.54±8.67	-0.4715	
85	Ciclev10001978m.g	BES1	68.82±2	86.73±4.42	68.6±3.15	77.47±5.2	-0.4877	
86	Ciclev10019816m.g	bHLH	38.33±0.9	46.51±1.27	47.07±3.39	35.61±0.66	-0.4924	
87	Ciclev10000188m.g	Nin-like	4.92±0.2	10.52±0.65	5.02±1.03	7.94±0.24	-0.5169	
88	Ciclev10027178m.g	MYB	0.02±0.02	0.33±0.23	2.51±0.43	0.4±0.05	-0.5201	
89	Ciclev10032012m.g	WRKY	9.56±4.58	24.61±2.78	23.6±2.55	62.03±2.95	-0.5288	
90	Ciclev10015261m.g	EIL	11.76±0.74	14.72±1.39	15.64±2.43	11.07±0.52	-0.5436	

91	Ciclev10012152m.g	MYB	12.18±1.11	14.8±0.81	93.03±15.77	45.51±2.35	-0.5456
92	Ciclev10001508m.g	TALE	0.01±0.01	0.38±0.08	0.99±0.24	2.2±0.22	-0.5489
93	Ciclev10023857m.g	ERF	0.27±0.18	0.23±0.00	2.74±0.22	2.48±0.72	-0.5502
94	Ciclev10002297m.g	C2H2	373.3±91.53	981.49±269.14	2483.93±162.91	514.63±68.93	-0.5614
95	Ciclev10020323m.g	bHLH	0.06±0.02	0.96±0.13	0.54±0.16	2.15±0.08	-0.5705
96	Ciclev10026197m.g	HD-ZIP	74.89±0.85	122.18±9.18	240.6±49.51	92.08±3.32	-0.5788
97	Ciclev10032490m.g	MIKC_MADS	7.51±0.5	19.37±2.17	32.47±0.69	61.91±2.39	-0.5857
98	Ciclev10018094m.g	Dof	5.98±0.52	15.71±0.28	36.59±9.38	9.78±0.19	-0.6037
99	Ciclev10005220m.g	ERF	11.94±0.39	47.44±4.74	54.56±2.43	9.34±0.63	-0.6129
100	Ciclev10012265m.g	MYB	2.16±0.4	5.75±0.35	20.43±4.13	7.69±1	-0.6195
101	Ciclev10012135m.g	bHLH	32.29±0.72	41.37±1.07	36.19±2.02	33.78±2.2	-0.6445
102	Ciclev10020967m.g	MYB	13.43±0.58	24.48±1.79	70.37±7.15	78.42±6.1	-0.6518
103	Ciclev10020053m.g	bHLH	10.82±1.4	47.46±1.32	24.13±2.17	21.55±2.64	-0.6773
104	Ciclev10025735m.g	bHLH	7.21±1.19	7.58±0.56	8.81±1.06	8.9±1.04	-0.6855
105	Ciclev10026107m.g	MYB	44.08±5.72	84.56±1.37	176.67±20.99	86.27±8.35	-0.6883
106	Ciclev10009761m.g	WRKY	10.83±1.22	54.67±2.76	64.08±9.52	14.79±0.98	-0.6907
107	Ciclev10001159m.g	bZIP	57.26±1.57	69.76±9.42	96.06±5.34	71.54±3.32	-0.7168
108	Ciclev10005701m.g	ERF	58.91±4.37	124.94±18.52	96.2±11.37	158.32±12	-0.7401
109	Ciclev10021877m.g	HD-ZIP	30.91±3.83	240.69±35.99	121.58±36.93	110.65±9.44	-0.7508
110	Ciclev10026336m.g	Dof	4.62±0.69	59.88±6.96	97.56±13.31	25.68±2.58	-0.7555
111	Ciclev10032020m.g	bHLH	31.1±1.53	38.78±0.95	36.64±0.17	43.57±2.08	-0.7710
112	Ciclev10005629m.g	MYB	3.5±2.22	41.39±11.24	43.98±4.26	12.76±2.66	-0.7801
113	Ciclev10009361m.g	ERF	23.88±1.74	134.16±15.1	227.27±3.38	95.93±6.05	-0.8098
114	Ciclev10005658m.g	GATA	24.43±2.35	57.93±4.51	85.08±1.36	48.55±2.92	-0.8331
115	Ciclev10028908m.g	MYB	14.36±1	26.48±1.06	43.26±3.18	33.24±2.21	-0.8387
116	Ciclev10031946m.g	MYB	5.08±0.51	14.5±0.68	17.59±2.85	9.32±0.26	-0.8423
117	Ciclev10032059m.g	ERF	16.54±1.46	44.8±2.29	31.06±5.71	41.39±2.54	-0.8612
118	Ciclev10015049m.g	WRKY	46.36±1.18	80.37±3.91	67.16±1.6	64.23±2.87	-0.8813
119	Ciclev10025963m.g	bZIP	40.88±1.97	93.18±4.32	82.19±1.08	64.29±4.18	-0.8892
120	Ciclev10015893m.g	G2-like	46.76±0.92	73.38±0.14	65.77±2.5	75.11±4.78	-0.9213
121	Ciclev10026457m.g	MIKC_MADS	0.83±0.03	1.85±0.1	1.92±0.09	2.19±0.22	-0.9434
122	Ciclev10019585m.g	bHLH	8.17±0.68	24.62±1.97	28.85±3.35	23.5±1.07	-0.9828

123	Ciclev10004984m.g	G2-like	17.49±1.53	48.6±6.49	44.58±3.13	45.12±5.27	-0.9895	
TFs (No.124 - No.148) with low FPKM values were filtered out. (Conditions: The average of the FPKM value of any stage is 0, or the sum of the FPKM value of the four stages is less than 1)								
124	Ciclev10013851m.g	AP2	0±0	0±0	0±0	0±0		
125	Ciclev10013385m.g	bZIP	0.14±0.09	0±0	0.04±0.02	0.05±0.02		
126	Ciclev10032777m.g	bZIP	1.87±0.78	2.25±0.54	0±0	0.16±0.12		
127	Ciclev10013814m.g	Dof	0±0	0±0	0±0	0±0		
128	Ciclev10022244m.g	ERF	0±0	0±0	0±0	0±0		
129	Ciclev10033578m.g	ERF	0±0	0±0	0.04±0.04	0±0		
130	Ciclev10006861m.g	ERF	0±0	0±0	0.04±0.04	0.02±0.02		
131	Ciclev10017494m.g	ERF	0.32±0.1	0.07±0.03	0±0	0±0		
132	Ciclev10017910m.g	LBD	0.09±0.09	0.1±0.05	0.04±0.04	0.09±0.05		
133	Ciclev10022371m.g	LBD	1.86±0.23	0.16±0.02	0±0	0±0		
134	Ciclev10021992m.g	LBD	0.29±0.04	0.12±0.04	0.21±0.05	0.21±0.11		
135	Ciclev10002471m.g	MIKC_MADS	0.15±0.1	0±0	0±0	0±0		
136	Ciclev10006752m.g	MYB	0±0	0±0	0.03±0.03	0.04±0.02		
137	Ciclev10003958m.g	MYB	0±0	0.02±0.02	0.06±0.06	0±0		
138	Ciclev10013466m.g	MYB	3.01±0.48	0.7±0.07	0.14±0.09	0±0		
139	Ciclev10033941m.g	MYB	0.11±0.04	0.33±0.11	0.11±0.07	0.1±0.03		
140	Ciclev10017764m.g	MYB	0±0	0±0	0±0	0±0		
141	Ciclev10026023m.g	MYB	0.06±0.04	0.16±0.1	0.05±0.03	0.17±0.07		
142	Ciclev10023756m.g	MYB	0±0	0±0	0±0	0±0		*MYBF2*
143	Ciclev10008921m.g	MYB	0.16±0.06	0.1±0.08	1±0.33	0±0		
144	Ciclev10013455m.g	MYB	0±0	0±0	0±0	0.07±0.07		*ruby1*
145	Ciclev10024588m.g	NAC	0±0	0±0	0±0	0±0		
146	Ciclev10006623m.g	NAC	0.04±0.02	0.04±0.02	0±0	0±0		
147	Ciclev10024257m.g	WRKY	0.27±0.13	0±0	0±0	0±0		
148	Ciclev10014984m.g	WRKY	0.08±0.04	0.39±0.06	0.09±0.05	0.07±0.04		

Table S3. Primers used in the present study.

Primer Name	Sequence (5' to 3')
**For promoter and gene cloning (into pMD19-T vector)**	
CitCHIL1-FL-FP	ATGGCCACTGAAGTTGTAATGG
CitCHIL1-FL-RP	CGGCTCATTTTGATAACTCAGCTG
CitCHI-FL-FP	ATGAATCCCTCACCGTCCGTCA
CitCHI-FL-RP	CGCGCTCATTTCATCTTATCACTAG
CitCHS1-FL-FP	ATGGTGACCGTCGATGAAGTTCG
CitCHS1-FL-RP	TTAAGCAGCGGCAACACTGTGG
CitCHS2-FL-FP	ATGGCAACCGTTCAAGAGATC
CitCHS2-FL-RP	TCAAGCTTTGATGGGGACACTG
CHIL1pro-FL-FP	GATTTCAAGACCATGACCATTACCC
CHIL1pro-FL-RP	CGGGGTAATCTTTAGTGGGAAATTATTGG
**For quantitative RT-PCR**	
CitCHIL1-Q-FP	GCAGCTCTGCGCTTTCTAAT
CitCHIL1-Q-RP	TCTTGAAGGACCTTAGCAAAGC
CitCHI-Q-FP	AATGCTGATGTCCCTGACCAAC
CitCHI-Q-RP	CAGTAGTGGCTAAGTGGCTTCC
CitCHIL2-Q-FP	TGAACTGCGAGAACAGAACCAA
CitCHIL2-Q-RP	CTCCTTTGCCTCCTCCTTTCG
CitFAP1-Q-FP	GCATACGCTCTGTACGGAGT
CitFAP1-Q-RP	GCTCAATCACAGATCCCTTAGG
CitFAP2-Q-FP	CAACGCTGTGAAGGATGTCT
CitFAP2-Q-RP	TAGGTGCAGTCCATAGCAAGGA
CitFAP3-Q-FP	CTGTCCACATTCCGAAGCATCT
CitFAP3-Q-RP	TGGCAACCCATCTCCTGAAATG
CitCHS1-Q-FP	GGCTTAGAGACCGCAGGAGAA
CitCHS1-Q-RP	TGAGTCAAACCAACCACACCAA
CitCHS2-Q-FP	TCACCGTCGAGACCGTTGT
CitCHS2-Q-RP	AACCTTCACACGATCAACCAAT
CitCHIL1-Qcds-FP	GCAACATCCCCTACTGCTGA
CitCHIL1-Qcds-RP	CCTTGCTCCACCCAAGTACC
AtCHIL-Q-FP	AGTTCACTGCGATCGGAGTT
AtCHIL-Q-RP	TCCTCAGCCAAACGATCTCT
AtEF1α-Q-FP	TGAGCACGCTCTTCTTGCTTTCA
AtEF1α-Q-RP	GGTGGTGGCATCCATCTTGTTACA
CitActin-FP	CATCCCTCAGCACCTTCC
CitActin-RP	CCAACCTTAGCACTTCTCC
CitRAV1-Q-FP	TGTTTCATGACGGTTTGAGTTGG
CitRAV1-Q-RP	GGGAAAGCAACAACACCACAA
CitERF32-Q-FP	TATGCTGGGATGGCGTGAAG
CitERF32-Q-RP	CCTGAAGAACCTTCCGTCCC
CitERF33-Q-FP	GCACAGGCTAGCTCAAGGAA
CitERF33-Q-RP	GGATTTCGGCCACCCATTTG
CitRAV1-cdsQ-FP	TCTTGTGCTGGAACGTCCTC
CitRAV1-cdsQ-RP	GCTCGTCGTTGTAAGTGTGC
**For subcellular localization (into 35S-eGFP vector)**	
CitCHIL1-GFP-FP	cggtacccggggatccATGGCCACTGAAGTTGTAATGG
CitCHIL1-GFP-RP	cgactctagaggatccTTTTGATAACTCAGCTGAGAGAG
CitCHI-GFP-FP	cggtacccggggatccATGAATCCCTCACCGTCC
CitCHI-GFP-RP	cgactctagaggatccTTTCATCTTATCACTAGTTACATTC
CitCHS1-GFP-FP	gagctcggtacccggggatccATGGTGACCGTCGATGAAGTTC
CitCHS1-GFP-RP	catgtcgactctagaggatccAGCAGCGGCAACACTGTGG
CitCHS2-GFP-FP	cggtacccggggatccATGGCAACCGTTCAAGAGATCAG
CitCHS2-GFP-RP	cgactctagaggatccAGCTTTGATGGGGACACTGTG
CitRAV1-GFP-FP	cggtacccggggatccATGAAAAGAGAAGAAATGGACGGA

CitRAV1-GFP-RP	cgactctagaggatccCGAAGCTCCAATCATTCTTTGT
CitERF32-GFP-FP	cggtacccggggatccATGGAGAGTTACAAAAAATCCCCA
CitERF32-GFP-RP	cgactctagaggatccGAAGTTCCAAATGGAGGTAGGCA
CitERF33-GFP-FP	cggtacccggggatccATGTCAAAAGCACTTATGGGTGG
CitERF33-GFP-RP	cgactctagaggatccAACGCACCACGGAGTCGG
**For recombinant protein constructs (into pET32a vector or pGEX-4t-1 vector)**	
CitCHIL1-His-FP	acctgagctcATGGCCACTGAAGTTGTAATGG
CitCHIL1-His-RP	cggcctcgagTTTTGATAACTCAGCTG
CitCHI-His-FP	acctgaattcATGAATCCCTCACCGTCCGTCA
CitCHI-His-RP	cggcctcgagTTTCATCTTATCACTAG
CitCHS1-His-FP	acctgaattcATGGTGACCGTCGATGAAGTTCG
CitCHS1-His-RP	aatactcgagAGCAGCGGCAACACTGTGG
CitCHS2-His-FP	cgacgaattcATGGCAACCGTTCAAGAGATC
CitCHS2-His-RP	attactcgagAGCTTTGATGGGGACACTG
CitRAV1-His-FP	gctgatatcggatccgaattcATGAAAAGAGAAGAAATGGACGGA
CitRAV1-His-RP	gtggtggtggtggtgctcgagCGAAGCTCCAATCATTCTTTGTT
CitERF32-His-FP	gctgatatcggatccgaattcATGGAGAGTTACAAAAAATCCCCA
CitERF32-His-RP	gtggtggtggtggtgctcgagGAAGTTCCAAATGGAGGTAGGCA
CitERF33-His-FP	gctgatatcggatccgaattcATGTCAAAAGCACTTATGGGTGG
CitERF33-His-RP	gtggtggtggtggtgctcgagAACGCACCACGGAGTCGG
CitERF33-GST-FP	gatctggttccgcgtggatccATGTCAAAAGCACTTATGGGT
CitERF33-GST-RP	gtcacgatgcggccgctcgagTCAAACGCACCACGGAGTCGG
**For Y2H assays (into pGADT7 or pGBKT7 vector)**	
CitCHIL1-BD-FP	aggaggacctgcatatgATGGCCACTGAAGTTGTAATGG
CitCHIL1-BD-RP	ggatccccgggaattcTCATTTTGATAACTCAGCTGAGAG
CitCHI-AD-FP	ggaggccagtgaattcATGAATCCCTCACCGTCC
CitCHI-AD-RP	tcatctgcagctcgagTCATTTCATCTTATCACTAGTTACA
CitCHS1-AD-FP	ggaggccagtgaattcATGGTGACCGTCGATGAAGT
CitCHS1-AD-RP	tcatctgcagctcgagTTAAGCAGCGGCAACACT
CitCHS2-AD-FP	ggaggccagtgaattcATGGCAACCGTTCAAGAGATCAGA
CitCHS2-AD-RP	tcatctgcagctcgagTCAAGCTTTGATGGGGACACTGT
**For BiFC assays (into p2YN and p2YC vectors)**	
CitCHIL1-2YN-FP	atttacgaacgatagttaattaaTATGGCCACTGAAGTTGTAATGGT
CitCHIL1-2YN-RP	acctcctccactagtggcgcgcccCTTTTGATAACTCAGCTGAGAGAGTATTG
CitCHI-2YN-FP	atttacgaacgatagttaattaaTATGAATCCCTCACCGTCCG
CitCHI-2YN-RP	acctcctccactagtggcgcgcccCTTTCATCTTATCACTAGTTACATTCAACA
CitCHS1-2YN-FP	atttacgaacgatagttaattaaTATGGTGACCGTCGATGAAGTTC
CitCHS1-2YN-RP	acctcctccactagtggcgcgcccCAGCAGCGGCAACACTGTG
CitCHS2-2YN-FP	atttacgaacgatagttaattaaTATGGCAACCGTTCAAGAGATCA
CitCHS2-2YN-RP	acctcctccactagtggcgcgcccCAGCTTTGATGGGGACACTGTG
CitRAV1-2YN-FP	atttacgaacgatagttaattaaTATGAAAAGAGAAGAAATGGACGGA
CitRAV1-2YN-RP	acctcctccactagtggcgcgcccCCGAAGCTCCAATCATTCTTTG
CitERF32-2YN-FP	atttacgaacgatagttaattaaTATGGAGAGTTACAAAAAATCCCCA
CitERF32-2YN-RP	acctcctccactagtggcgcgcccCGAAGTTCCAAATGGAGGTAGG
CitERF33-2YN-FP	atttacgaacgatagttaattaaTATGTCAAAAGCACTTATGGGTGG
CitERF33-2YN-RP	acctcctccactagtggcgcgcccCAACGCACCACGGAGTCG
**For LCI assays (into nLUC and cLUC vectors)**	
CitCHIL1-nLUC-FP	acgggggacgagctcggtaccATGGCCACTGAAGTTGTAATGGT
CitCHIL1-nLUC-RP	cgcgtacgagatctggtcgacTTTTGATAACTCAGCTGAGAGAGTATTG
CitCHI-nLUC-FP	acgggggacgagctcggtaccATGAATCCCTCACCGTCCG
CitCHI-nLUC-RP	cgcgtacgagatctggtcgacTTTCATCTTATCACTAGTTACATTCAACAG
CitCHS1-nLUC-FP	acgggggacgagctcggtaccATGGTGACCGTCGATGAAGTTC
CitCHS1-nLUC-RP	cgcgtacgagatctggtcgacAGCAGCGGCAACACTGTGG
CitCHS2-nLUC-FP	acgggggacgagctcggtaccATGGCAACCGTTCAAGAGATCA
CitCHS2-nLUC-RP	cgcgtacgagatctggtcgacAGCTTTGATGGGGACACTGTGC
AtCHS-nLUC-FP	acgggggacgagctcggtaccATGGTGATGGCTGGTGCTTC

AtCHS-nLUC-RP	cgcgtacgagatctggtcgacGAGAGGAACGCTGTGCAAGACG
CitCHIL1-cLUC-FP	tacgcgtcccggggcggtaccATGGCCACTGAAGTTGTAATGGT
CitCHIL1-cLUC-RP	acgaaagctctgcaggtcgacTCATTTTGATAACTCAGCTGAGAGAGT
CitCHI-cLUC-FP	tacgcgtcccggggcggtaccATGAATCCCTCACCGTCCG
CitCHI-cLUC-RP	acgaaagctctgcaggtcgacTCATTTCATCTTATCACTAGTTACATTCAA
CitCHS1-cLUC-FP	tacgcgtcccggggcggtaccATGGTGACCGTCGATGAAGTTC
CitCHS1-cLUC-RP	acgaaagctctgcaggtcgacTTAAGCAGCGGCAACACTGTG
CitCHS2-cLUC-FP	tacgcgtcccggggcggtaccATGGCAACCGTTCAAGAGATCA
CitCHS2-cLUC-RP	acgaaagctctgcaggtcgacTCAAGCTTTGATGGGGACACTG
AtCHS-cLUC-FP	tacgcgtcccggggcggtaccATGGTGATGGCTGGTGCTTC
AtCHS-cLUC-RP	acgaaagctctgcaggtcgacTTAGAGAGGAACGCTGTGCAAG
**For overexpression (into pBI121 vector)**	
CitCHIL1-pBI-FP	ggactctagaggatccATGGCCACTGAAGTTGTAATGG
CitCHIL1-pBI-RP	gaccacccggggatccTTTGATAACTCAGCTGAGAGAG
CitRAV1-pBI-FP	ggactctagaggatccATGAAAAGAGAAGAAATGGACGGA
CitRAV1-pBI-RP	gaccacccggggatccCGAAGCTCCAATCATTCTTTGT
CitERF32-pBI-FP	ggactctagaggatccATGGAGAGTTACAAAAAATCCCCA
CitERF32-pBI-RP	gaccacccggggatccGAAGTTCCAAATGGAGGTAGGCA
CitERF33-pBI-FP	ggactctagaggatccATGTCAAAAGCACTTATGGGTGG
CitERF33-pBI-RP	gaccacccggggatccAACGCACCACGGAGTCGG
**For VIGS (into TRV2 vector)**	
CitCHIL1-TRV2	gcttgaattcCACCACCAAGCCGTTGAATC
CitCHIL1-TRV2	ggaaggtaccAACGATTTTCTCAAGGGCAG
**For dual-luciferase assays (into LUC vector or SK vector)**	
CHIL1pro-LUC-FP	ACTCGGATCCGATTTCAAGACCATGACCAT
CHIL1pro-LUC-RP	GCGGTCCATGGTAATCTTTAGTGGGAAATTA
AN20/AN1-SK-FP	cgggctgcaggaattcATGGCGGAGGAGCACAGATTC
AN20/AN1-SK-RP	cgggccccccctcgagTCAAATCTTCTCGAGCTTCTCCGC
NAC47-SK-FP	cgggctgcaggaattcATGGTTTGCATAAAGAACCCGG
NAC47-SK-RP	cgggccccccctcgagTCATCCTTGAAACTGAAGATGTGGG
UNE12-SK-FP	cgggctgcaggaattcATGTCTACCACTGGCAATGGC
UNE12-SK-RP	cgggccccccctcgagCTATGAAGGGGTGCTCGATTCAGG
MYC2-SK-FP	cgggctgcaggaattcATGACGGACTACCGGTTACCTTCA
MYC2-SK-RP	cgggccccccctcgagTTATTGGGTATCTCCAACTTTGGC
ERF#74-SK-FP	cgggctgcaggaattcATGGAGGATTGCGAAGCT
ERF#74-SK-RP	cgggccccccctcgagTTAATCTGCAGTGATGGATGAGGA
bHLH162-SK-FP	tcccccgggctgcaggaattcATGTTCAAGAAATTAAGCATAGCTTATCC
bHLH162-SK-RP	ggtaccgggccccccctcgagTTAGAACTCCCACAATTCTGGATTG
WRKY70-SK-FP	cgggctgcaggaattcATGGGCACTCTTTGTTCAGAGA
WRKY70-SK-RP	cgggccccccctcgagCTAAAAGATTCCGCTGTCATCA
WRKY46-SK-FP	cgggctgcaggaattcATGAAAAGGTCGACGATGGATAGGG
WRKY46-SK-RP	cgggccccccctcgagCTAGAAGCAATCCAAGTCGTCAAAC
SEPALLATA1-SK-FP	cgggctgcaggaattcATGGGAAGAGGGAGGGTTGAG
SEPALLATA1-SK-RP	cgggccccccctcgagTCACAGCATCCATCCTGGG
HSFA3-SK-FP	cgggctgcaggaattcATGGACAAAGAGGAACAAAACTAC
HSFA3-SK-RP	cgggccccccctcgagTTACAGTAGGGGTGATTCAT
DOF1.2-SK-FP	cgggctgcaggaattcATGGAGAAAAAATGGAAACCAGACG
DOF1.2-SK-RP	cgggccccccctcgagTCAAGGTCTTGAATAGAGCTCAAA
ERF#25-SK-FP	cgggctgcaggaattcATGTCTGTAACGACACAAACAC
ERF#25-SK-RP	cgggccccccctcgagTTACAATTCTAAACAAGACCCCAG
GTE12-SK-FP	cgggctgcaggaattcATGATTGCTACTGAAGCTGTAA
GTE12-SK-RP	cgggccccccctcgagTCATGAAAAAATTTCTCCCTCCTCG
ERF#16-SK-FP	cgggctgcaggaattcATGGACATATTATTCGGCCAAG
ERF#16-SK-RP	cgggccccccctcgagTCAAATTGAATGACTCCACAACGAC
CitABF2-SK-FP	tcccccgggctgcaggaattcATGGGAACACAGACAATGGGAT
CitABF2-SK-RP	ggtaccgggccccccctcgagTCAGAAGGGTGCTGAGCCTG

CitAGL6-SK-FP	tcccccgggctgcaggaattcATGGGGAGAGGAAGAGTGGAGC
CitAGL6-SK-RP	ggtaccgggccccccctcgagTCAAAGGACCCATCCTTGGAT
CitERF32-SK-FP	tcccccgggctgcaggaattcATGGAGAGTTACAAAAAATCCCCA
CitERF32-SK-RP	ggtaccgggccccccctcgagTCAGAAGTTCCAAATGGAGGTAGG
CitERF33-SK-FP	tcccccgggctgcaggaattcATGTCAAAAGCACTTATGGGTGG
CitERF33-SK-RP	ggtaccgggccccccctcgagTCAAACGCACCACGGAGTCG
CitAP2-5-SK-FP	tcccccgggctgcaggaattcATGGAGGTTCCTCAAGACCCTC
CitAP2-5-SK-RP	ggtaccgggccccccctcgagCTAAGCATCAGTCCATGCAGCA
CitRF2b-SK-FP	tcccccgggctgcaggaattcATGGAAAGCCAAGATCCCCC
CitRF2b-SK-RP	ggtaccgggccccccctcgagTCAAAATGTGCTGCTGCTTTCA
CitMYB44-SK-FP	tcccccgggctgcaggaattcATGAAAATGGAACCACAAACGG
CitMYB44-SK-RP	ggtaccgggccccccctcgagTTAGTTTTTAATCCTACCAGCCTCAT
CitMADS1-SK-FP	tcccccgggctgcaggaattcATGGCGTTTCCAAATGAATTAGC
CitMADS1-SK-RP	ggtaccgggccccccctcgagTTAAACTAACTGAAGGGCCATCTG
CitMYB108-SK-FP	tcccccgggctgcaggaattcATGCACACAATGAGAGCAGCAA
CitMYB108-SK-RP	ggtaccgggccccccctcgagTTACTCCCTAAGCTGCCATATGTC
CitAZF2-SK-FP	tcccccgggctgcaggaattcATGGAGAAGCACAAGTGTAGGCT
CitAZF2-SK-RP	ggtaccgggccccccctcgagTCACCGTTTGATGTGGTTTACAA
CitRAV1-SK-FP	tcccccgggctgcaggaattcATGAAAAGAGAAGAAATGGACGGA
CitRAV1-SK-RP	ggtaccgggccccccctcgagTTACGAAGCTCCAATCATTCTTTG
CitGT-2-SK-FP	tcccccgggctgcaggaattcATGGAAAAACGAACTGGCGA
CitGT-2-SK-RP	ggtaccgggccccccctcgagTTAATCCTCAATCTTGGCAGCA
CitATHB-13-SK-FP	tcccccgggctgcaggaattcATGTCTTGCAATGGGATGGC
CitATHB-13-SK-RP	ggtaccgggccccccctcgagTCAACTGAAATTGTGTTGCTCCA
CitbHLH130-SK-FP	tcccccgggctgcaggaattcATGGATTCAAGTACTAATCATAATTATCATCA
CitbHLH130-SK-RP	ggtaccgggccccccctcgagTCAAACAATTTGATTCTCGACTGG
CitREVEILLE6-SK-FP	tcccccgggctgcaggaattcATGGTATCAAAAATCCCGAACCC
CitREVEILLE6-SK-RP	ggtaccgggccccccctcgagCTATTGGCCACCACCGAGG
CitDof5.2-SK-FP	tcccccgggctgcaggaattcATGCGGCAAGAAAGTAAAGATCC
CitDof5.2-SK-RP	ggtaccgggccccccctcgagTCAAGTGCTTTCATGGAAGTTGA
CitbHLH74-SK-FP	tcccccgggctgcaggaattcATGGGTGCTGGTGACAATGATG
CitbHLH74-SK-RP	ggtaccgggccccccctcgagTTAAAGCTCCGACTTCAAGCG
CitSMH3-SK-FP	tcccccgggctgcaggaattcATGGGAAATCAGAAGCAAAAGTG
CitSMH3-SK-RP	ggtaccgggccccccctcgagTCAAGCCAAAAGAACAATTTCACC
CitGATA16-SK-FP	tcccccgggctgcaggaattcATGATGGATCCGAGTGACAAAGG
CitGATA16-SK-RP	ggtaccgggccccccctcgagCTAAGCATAAACGGAACCATAAGATAA
CitERF48-SK-FP	tcccccgggctgcaggaattcATGGTTAATAGCAGACACAGTGTTATCC
CitERF48-SK-RP	ggtaccgggccccccctcgagTTAAATAGCAACAAGGTTATCCGACG
**For Y1H assays (into pAbAi vector or pGADT7 vector)**	
CHIL1proA-pAbAi-FP	AAAAGCTTGAATTCGAGCTCGATTTCAAGACCATGACCATTACC
CHIL1proA-pAbAi-RP	ATGCCTCGAGGTCGACGTAGTCTATATTGAGGTTAATGTCC
CHIL1proB-pAbAi-FP	AAAAGCTTGAATTCGAGCTCCAATGTAGCCTCATGAGTCAACAGC
CHIL1proB-pAbAi-RP	ATGCCTCGAGGTCGACCTAGAGTGCTACCATCCAAAGG
CHIL1proC-pAbAi-FP	AAAAGCTTGAATTCGAGCTCAGGGACTTACTTTAATTTTAAAACC
CHIL1proC-pAbAi-RP	ATGCCTCGAGGTCGACGGTAATCTTTAGTGGGAAATTATTG
AN20/AN1-AD-FP	ggaggccagtgaattcATGGCGGAGGAGCACAGATTC
AN20/AN1-AD-RP	tcatctgcagctcgagTCAAATCTTCTCGAGCTTCTCCGC
NAC47-AD-FP	ggaggccagtgaattcATGGTTTGCATAAAGAACCCGG
NAC47-AD-RP	tcatctgcagctcgagTCATCCTTGAAACTGAAGATGTGGG
UNE12-AD-FP	ggaggccagtgaattcATGTCTACCACTGGCAATGGC
UNE12-AD-RP	tcatctgcagctcgagCTATGAAGGGGTGCTCGATTCAGG
MYC2-AD-FP	ggaggccagtgaattcATGACGGACTACCGGTTACCTTCA
MYC2-AD-RP	tcatctgcagctcgagTTATTGGGTATCTCCAACTTTGGC
ERF#74-AD-FP	ggaggccagtgaattcATGGAGGATTGCGAAGCT
ERF#74-AD-RP	tcatctgcagctcgagTTAATCTGCAGTGATGGATGAGGA
bHLH162-AD-FP	ggaggccagtgaattcATGTTCAAGAAATTAAGCATAGCT
bHLH162-AD-RP	tcatctgcagctcgagTTAGAACTCCCACAATTCTGGA

WRKY70-AD-FP	ggaggccagtgaattcATGGGCACTCTTTGTTCAGAGA
WRKY70-AD-RP	tcatctgcagctcgagCTAAAAGATTCCGCTGTCATCA
WRKY46-AD-FP	ggaggccagtgaattcATGAAAAGGTCGACGATGGATAGGG
WRKY46-AD-RP	tcatctgcagctcgagCTAGAAGCAATCCAAGTCGTCAAAC
SEPALLATA1-AD-FP	ggaggccagtgaattcATGGGAAGAGGGAGGGTTGAG
SEPALLATA1-AD-RP	tcatctgcagctcgagTCACAGCATCCATCCTGGG
HSFA3-AD-FP	ggaggccagtgaattcATGGACAAAGAGGAACAAAACTAC
HSFA3-AD-RP	tcatctgcagctcgagTTACAGTAGGGGTGATTCAT
DOF1.2-AD-FP	ggaggccagtgaattcATGGAGAAAAAATGGAAACCAGACG
DOF1.2-AD-RP	tcatctgcagctcgagTCAAGGTCTTGAATAGAGCTCAAA
ERF#25-AD-FP	ggaggccagtgaattcATGTCTGTAACGACACAAACAC
ERF#25-AD-RP	tcatctgcagctcgagTTACAATTCTAAACAAGACCCCAG
GTE12-AD-FP	ggaggccagtgaattcATGATTGCTACTGAAGCTGTAA
GTE12-AD-RP	tcatctgcagctcgagTCATGAAAAAATTTCTCCCTCCTCG
ERF#16-AD-FP	ggaggccagtgaattcATGGACATATTATTCGGCCAAG
ERF#16-AD-RP	tcatctgcagctcgagTCAAATTGAATGACTCCACAACGAC
